# A chromosome-scale reference genome and integrative transcriptome provide insight into tissue- and stress-specific responses in tetraploid sainfoin *(Onobrychis viciifolia)*

**DOI:** 10.1007/s00425-026-05021-y

**Published:** 2026-05-14

**Authors:** Cuong V. Nguyen, Dustin Cram, Halim Song, Rodrigo Ortega Polo, Hari Poudel, Bill Biligetu, Kimberley Burton Hughes, Surya Acharya, David Konkin, Stacy D. Singer

**Affiliations:** 1 Agriculture and Agri-Food Canada, Lethbridge Research and Development Centre, Lethbridge, AB T1J 4B1 Canada; 2 National Research Council of Canada, Aquatic Crop Resource Development, Saskatoon, SK S7N 0W9 Canada; 3 Department of Plant Sciences, College of Agriculture and Bioresources, University of Saskatchewan, Saskatoon, SK S7N 5A8 Canada

**Keywords:** Abiotic stress response, Forage legume, Haplotype-resolved polyploid genome, Sainfoin, Transcriptomics

## Abstract

**Main conclusion:**

**A haplotype-resolved, chromosome-scale AAC Mountainview sainfoin genome sequence along with organelle assemblies, methylome, and comparative tissue/stress-specific transcriptomes provide a solid foundation to support downstream sainfoin breeding efforts.**

**Abstract:**

Sainfoin (*Onobrychis viciifolia* Scop.) is an outcrossing, perennial forage crop valued for its leguminous nature, nutritional quality, palatability, and production of condensed tannins, which reduce the incidence of pasture bloat in ruminants and improve feed efficiency. However, despite its potential, there remains a paucity of research and breeding efforts focusing on this species. In this study, we report the generation of a haplotype-resolved, chromosome-scale reference genome sequence for a genotype of the tetraploid sainfoin cultivar AAC Mountainview, which was assembled using PacBio HiFi, Oxford Nanopore, Illumina short read, and Hi-C data. The 2.36 Gb assembly resolves all 28 pseudochromosomes, corresponding to 4 haplotypes of the 7 base chromosomes, with high contiguity and gene completeness, as well as reference-grade long terminal repeat (LTR) assembly index (LAI) scores across all haplotypes. Nanopore-based methylation profiling revealed typical gene body CG methylation, as well as high levels of transposable element methylation. We annotated 117,890 high-confidence protein-coding genes and identified large tandem arrays of rDNA localized on unanchored but chromosome-associated scaffolds. Mitochondrial and chloroplast genomes were also successfully assembled for this genotype. Furthermore, tissue- and stress-specific transcriptomic profiling revealed both shared and distinct gene expression responses across tissue and stress types. Allele-specific expression analysis showed largely balanced haplotype activity, with subtle but consistent shifts in allelic dominance under stress. The data provided in this study offer a valuable resource for downstream breeding endeavors in this promising forage crop.

**Supplementary Information:**

The online version contains supplementary material available at 10.1007/s00425-026-05021-y.

## Introduction

Sainfoin (*Onobrychis viciifolia* Scop.; Fabaceae) is a leguminous perennial forage crop recognized for its nutritional quality and palatability (Sheppard et al. 2019). It comprises two types, including the ‘common’ type (*O. sativa* var. *communis* Ahlefed; originating from central Europe), which does not re-flower after the first cut, and the ‘giant’ type (*O. sativa* var. *bifera* Hort.; originating from the Middle East), which re-flowers during regrowth (Poudel et al. 2023). While sainfoin was historically cultivated in Europe, Asia, and North America for hundreds of years (e.g., Mora-Ortiz and Smith 2018), its production has waned substantially over the last century due to the adoption of intensive farming methods and the overwhelming implementation of higher-yielding forage species such as alfalfa (*Medicago sativa* L.; Hayot Carbonero et al. 2011). However, in recent years it has once again been gaining renewed attention as a sustainable forage crop for semi-arid environments, including the Canadian prairies (e.g., Sheppard et al. 2019; Poudel et al. 2023).

At least part of this interest derives from the fact that sainfoin synthesizes condensed tannins (CTs; also known as proanthocyanidins) in a range of tissues, including leaves and stems (Wang et al. 2015a), which is in contrast to alfalfa, which only produces CTs at appreciable levels in its seed coats (Goplen et al. 1980). In ruminants, these CTs bind proteins in the acidic rumen and then release them in the higher pH abomasum and small intestines for digestion and absorption (Mueller-Harvey et al. 2019). This leads to a reduced incidence of pasture bloat (Min et al. 2006), improved dietary N utilization, and decreased ammonia emissions from livestock (Mueller-Harvey 2006). Furthermore, CTs in forages have also been shown to provide anthelmintic effects (Desrues et al. 2016) and may reduce methane emissions from the ruminants that consume them (Hatew et al. 2016).

While these characteristics are incredibly beneficial, sainfoin tends to exhibit at least somewhat reduced forage yields compared to alfalfa, as well as poor persistence and regrowth after harvesting or grazing (e.g., Lauriault and Marsalis 2024). Furthermore, although sainfoin can tolerate a certain extent of water-deficit due to its deep tap root (Poudel et al. 2023), it appears to be less resilient to drought than alfalfa (e.g., Biligetu et al. 2014, 2021), and substantial improvements are still needed in this area. In addition, sainfoin is particularly sensitive to waterlogging/flooding (Heinrichs 1970), which limits its production area substantially. To date, breeding efforts have led to some improvement in these, and other, traits in sainfoin (Acharya 2015; Monirifar 2022; Poudel and Acharya 2022); however, research in this species has been very sparse compared to other forage legumes such as alfalfa and clovers (*Trifolium* spp.) (Poudel et al. 2023), and there remains a relative scarcity of molecular information to guide breeding endeavors. This lack of genomic resources has restricted the mechanistic dissection of traits such as CT biosynthesis, as well as the improvement of persistence under grazing, regrowth capacity, and tolerance to abiotic stresses, for example.

Although a genome sequence for an unspecified ‘common’ type cultivar of sainfoin was recently released (He et al. 2024), most modern cultivars are derived from both ‘common’ and ‘giant’ types (Mora-Ortiz and Smith 2018), and as such, the genetic background of this cultivar likely differs substantially from those that are more widely grown today. To provide a more representative reference sequence for sainfoin cultivars commonly cultivated in Europe and North America, we endeavored to provide a genome sequence for a genotype of the tetraploid (2*n* = 4*x* = 28) sainfoin cultivar AAC Mountainview, which was developed in Canada from a broad range of cultivars/germplasm for its improved persistence in alfalfa mixtures, increased regrowth after harvest, and superior forage yield (Acharya 2015; Poudel et al. 2023). The fact that sainfoin is a genetically complex, obligately outcrossing and highly heterozygous species (Kempf et al. 2015) complicates this process. However, using a combination of PacBio HiFi and Oxford Nanopore long reads, Illumina short reads, and Hi-C data together with short- and long-read transcript evidence from diverse tissues allowed for the generation of a highly contiguous, haplotype-aware, well-annotated reference genome for this genotype. Chloroplast and mitochondrial genomes were also assembled, providing complete organelle references for future phylogenetic and functional studies. We also profiled genome-wide DNA methylation and investigated tissue-specific gene expression, preferential allele expression, and abiotic stress response at the transcriptional level. The availability of a haplotype-resolved reference genome, along with extensive gene annotations and expression data, from a widely cultivated sainfoin cultivar creates new opportunities for downstream molecular breeding, genome editing, pan-genome evaluations, and functional genetic studies in sainfoin to facilitate its agronomic improvement**.**

## Materials and methods

### Plant genotype and growth conditions

All genome and RNA sequencing was carried out using plants derived from a single genotype (propagated vegetatively) of *O. viciifolia* cultivar AAC Mountainview (Acharya 2015). To preserve and amplify the genotype throughout the experiment, vegetative propagation was used through the production of rooted stem cuttings. Plants were maintained under controlled greenhouse conditions with a 16 h/8 h photoperiod and approximately 22/18 °C day/night temperatures. For drought treatment, volumetric soil moisture contents were first brought to approximately 50%, after which time water was withheld. Soil moisture contents were measured using a ML3 ThetaKit soil moisture meter with 6 cm probes (Hoskin Scientific Ltd., Burnaby, BC, Canada). For waterlogging treatment, volumetric soil moisture levels were similarly brought to approximately 50%, after which time pots were submerged in water to ¾ pot height for the remainder of the treatment. Normally watered plants were used as controls.

## Illumina, Oxford Nanopore, and PacBio HiFi whole-genome sequencing

For Oxford Nanopore and Illumina sequencing, high-molecular-weight genomic DNA was extracted from young leaf tissue of our selected AAC Mountainview genotype using a salting-out based method (Doyle and Doyle 1987). Nanopore libraries were prepared using the Ligation Sequencing Kit V14 (SQK-LSK114) according to the manufacturer’s recommendations (Oxford Nanopore Technologies, Oxford, UK) and sequenced using R10.4.1 flow cells (Oxford Nanopore Technologies). Nanopore basecalling was completed using Dorado version 0.9.6 (https://github.com/nanoporetech/dorado) in duplex mode using the sup model and including 5-methylcytosine (5mC), 5-hydroxymethylcytosine (5hmC) and N6-methyladenine (6 mA) modifications. Short-read Illumina data were generated by a service provider on an Illumina NovaSeq 6000 platform (150 bp paired-end reads; Illumina Inc., San Diego, CA, USA) from libraries prepared using Novogene’s standard Illumina DNA library construction workflow (Novogene Corporation Inc., Sacramento, CA, USA). Reads were adapter-trimmed and quality-filtered using fastp v0.23 (Chen et al. 2018).

For PacBio HiFi sequencing, dark-treated (53 h) young leaf tissue was harvested and flash-frozen in liquid nitrogen. Plant nuclei were isolated using the LN2 protocol provided by PacBio (https://www.pacb.com/wp-content/uploads/Procedure-checklist-Isolating-nuclei-from-plant-tissue-using-LN2-disruption.pdf), and high-molecular-weight DNA was extracted using the Nanobind Plant Nuclei kit (Pacific Biosciences, Menlo Park, CA, USA). DNA length, quality and concentration were determined by pulsed field gel electrophoresis and Qubit Flex fluorometry (Thermo Fisher Scientific Inc., Waltham, MA, USA). Libraries were prepared using the SMRTbell Express Template Prep Kit 2.0 (Pacific Biosciences) and sequenced on a PacBio Revio platform (Pacific Biosciences) by a service provider (Novogene Corporation) to generate HiFi reads.

## Hi-C sequencing

Young leaf tissue was harvested from the selected AAC Mountainview genotype, flash-frozen in liquid nitrogen, and placed at -80˚C. Two Hi-C chromatin interaction libraries were prepared using the Arima-HiC kit with DpnII and HinfI (Arima Genomics, Inc., Carlsbad, CA, USA) and Phase Genomics kit with DpnII, DdeI, MseI, and HinfI (Phase Genomics, Inc., Seattle, WA, USA) across separate experiments. Sequencing was carried out on an Illumina NovaSeq 6000 platform in-house and by Phase Genomics, respectively.

## Estimation of genome size and ploidy

21-mer frequencies were computed from PacBio HiFi reads using KMC v3 (Kokot et al. 2017), zero-count entries removed, and genome size, repeat content, k-mers, and sequencing error rate were determined using GenomeScope v2.0 (Ranallo-Benavidez et al. 2020) with ploidy set to 4. For ploidy assessment, k-mer counts and allele multiplicity patterns were analyzed with Smudgeplot v0.2.5 (Ranallo-Benavidez et al. 2020). Coverage cutoffs were determined automatically with the cutoff function (L = 30, U = 4700).

## Genome assembly and scaffolding

PacBio HiFi, Nanopore duplex and Nanopore simplex reads were filtered for minimal lengths of 1,000, 1,000 and 20,000 bp, and minimal quality scores of 20, 20 and 10, respectively. Verkko was used for the assembly (Rautiainen et al. 2023) using PacBio HiFi, Oxford Nanopore and Hi-C data. Duplex Nanopore reads were specified as high accuracy (-hifi) along with PacBio HiFi reads. Simplex reads were only used for graph traversal (-nano). The assembly graph was inspected using Bandage (Wick et al. 2015). Hi-C reads were trimmed using Cutadapt (Martin 2011) with the flag -u 5 and aligned, filtered and combined using the Arima Genomics mapping pipeline (https://github.com/ArimaGenomics/mapping_pipeline).

Scaffolding was conducted with HapHiC using the flag –RE GATC, GANTC (Zeng et al. 2024). Scaffolds were manually curated with Juicebox (Robinson et al. 2018). Haplotigs and small unscaffolded contigs were reviewed for misassemblies and reassigned when supported by Hi-C contact maps and assembly graph contiguity. Remaining repetitive sequences (~ 200 Mb) were assigned to chromosomal groups based on the assembly graph (chr2un to chr7un). Illumina polishing of the assembly was completed with Pilon (Walker et al. 2014). Completeness and contiguity were assessed using BUSCO v5.3.2 (embryophyta_odb10, fabales_odb10; Simao et al. 2015).

## RNA-Seq and Iso-Seq

Leaf, stem, root, and flower tissues were harvested from plants approximately 3 months after cutting, when flowering is typically reached under our greenhouse conditions. Tissues were harvested from 4 biological replicate plants of the same AAC Mountainview genotype used for genome sequencing, each derived from independent clonal plants propagated from stem cuttings. Nine tissue types were collected and flash-frozen in liquid nitrogen, including emerging leaves (still tightly folded), developing leaves (open, but small), first fully expanded leaves (first from the shoot tip), third fully expanded leaves (third from the shoot tip), stems (the entirety of the third internode from the shoot tip), floral buds (unopened), open flowers, roots from the half nearest of the crown, and roots from the half closest to the root tips. Similarly, fully expanded leaves (third from the shoot tip) were also harvested from plants treated with drought and waterlogging, respectively, for 7 days, at which point plants were just beginning to exhibit signs of stress (drying leaves in the case of drought and chlorosis in the case of waterlogging). For drought, tissue was harvested when soil moisture levels were between 2.6% and 4.9% (compared to approximately 50% for well-watered plants). Harvesting of stressed and non-stressed tissues was carried out at the same time. Total RNA was extracted from all samples using the Sigma Spectrum Total RNA kit (protocol A) with on-column DNase treatment (Sigma-Aldrich Inc., St. Louis, MO, USA). RNA was further purified using the Norgen RNA Clean-up and Concentration kit (Norgen Biotek Corp., Thorold, ON, Canada) according to the manufacturer’s instructions. RNA quality, purity, and quantity were confirmed by agarose gel electrophoresis, as well as using a NanoDrop 8000 spectrophotometer (Thermo Fisher Scientific), Qubit Flex fluorometer, and Agilent 2100 Fragment Analyzer (Agilent Technologies Inc., Santa Clara, CA, USA).

For both RNA-Seq and Iso-Seq, library preparation and sequencing were carried out by a service provider (Novogene Corporation). In the case of RNA-Seq, stranded libraries were prepared using Novogene’s RNA-Seq library construction workflow and sequencing (150 bp paired-end reads) was carried out on an Illumina NovaSeq 6000 platform. Reads were quality-filtered and trimmed with fastp v0.23 (Chen et al. 2018), then aligned to our AAC Mountainview genome sequence using HISAT2 v2.2.1 (Kim et al. 2019). Gene-level counts were quantified with StringTie v2.2.1 (Pertea et al. 2015). For Iso-Seq, equimolar ratios of total RNA from a single sample of each tissue type were combined and sequenced. A library was prepared using the Kinnex Full-Length RNA kit (Pacific Biosciences) and sequenced on a PacBio Revio system to generate HiFi reads. Raw HiFi BAMs were processed with the PacBio Iso-Seq v3 toolchain (https://github.com/PacificBiosciences/IsoSeq).

## Transposable element annotation

Transposable elements (TEs) were annotated using RepeatMasker v4.1.2 and the EDTA v2.2.2 pipeline (Ou et al. 2019), incorporating a custom repeat library derived from the genome assembly itself, generated de novo using EDTA through the structural discovery of long terminal repeat (LTR) retrotransposons, terminal inverted repeat/miniature inverted-repeat (TIR/MITE) transposable elements, and Helitrons, followed by classification and whole-genome masking. TE distribution and classification were summarized, and genome contiguity in repetitive regions was evaluated using the LTR assembly index (LAI) with LTR_retriever (Ou and Jiang 2018).

## Gene and non-coding RNA prediction and functional annotation

Gene models were predicted using BRAKER3 (Gabriel et al. 2024) in protein-RNA mode with Fabales proteins from OrthoDB (Zdobnov et al. 2021), incorporating PacBio Iso-Seq data from 9 tissues in one run and Illumina RNA-Seq data from 9 tissues and 2 stress conditions in a second run. The results were combined using TSEBRA (Gabriel et al. 2021). Gene model quality was assessed with BUSCO (Benchmarking Universal Single-Copy Orthologs) v5.4.3 (fabales_odb10). Functional annotation employed DIAMOND BLASTP (Buchfink et al. 2021) searches against NCBI nr (https://www.ncbi.nlm.nih.gov/), Swiss-Prot (Boutet et al. 2007), and *Arabidopsis thaliana* TAIR (Rhee et al. 2003) databases. Domain predictions and orthology assignments were carried out using InterProScan v5.52 (Jones et al. 2014) and eggNOG-mapper v2.1.9 (Cantalapiedra et al. 2021), with the taxonomic scope restricted to plants. Gene ontology (GO) terms and Pfam domains from eggNOG-mapper were integrated into the comprehensive annotation set. Gene structure statistics, including exon number per transcript, isoform number per gene, and average exon and intron lengths, were calculated directly from the final GFF3 annotation by summarizing exon, intron, and transcript features across each chromosome. Sequences encoding ribosomal RNAs (rRNAs), transfer RNAs (tRNAs), and microRNAs (miRNAs) were identified using barrnap (https://github.com/tseemann/barrnap), tRNAscan-SE v2.0 (Chan et al. 2021), and Infernal using Rfam covariance models (Nawrocki and Eddy 2013).

## DNA methylation detection and profiling

Genome-wide DNA methylation profiles were generated from Oxford Nanopore reads. The reads were aligned to the reference genome using the Dorado aligner (Oxford Nanopore Technologies), which retains modification tags. Modification frequency data were summarized using modkit pileup to obtain per-site counts of modified and unmodified bases in BEDMethyl format. These files were split by motif and converted to bigWig format for downstream analysis and visualization. Metaplots of average methylation patterns across genes and TEs were produced using deepTools v3.5.5 (Ramírez et al. 2014).

## Orthology, phylogenomic, and synteny analysis

Orthology analysis was conducted using OrthoFinder v2.5.4 (Emms and Kelly 2019) with protein, coding sequence, and GFF3 files from sainfoin and 18 other reference angiosperms (14 of which were legumes and 4 were outgroups). Species trees from 233 high-occupancy orthogroups utilized MAFFT v7.490 (Katoh and Standley 2013) for alignment and FastTree2 (Price et al. 2010) for phylogenetic reconstruction, and were rooted using STRIDE (Emms and Kelly 2017) with *Arabidopsis*. Synteny blocks were identified with DIAMOND BLASTP (Buchfink et al. 2021) and MCScanX (Wang et al. 2012), and were analyzed for synonymous substitution rates (K_s_, K_a_, K_a_/K_s_) using TBtools v1.123 (Chen et al. 2023).

## Organelle genome assembly

Chloroplast and mitochondrial genomes were assembled and circularized using GetOrganelle (Jin et al. 2020), Tiara (Karlicki et al. 2022), and TIPPo (Xian et al. 2025) using Illumina and PacBio HiFi reads. Although our PacBio HiFi library was prepared from purified nuclei, low-level plastid/mitochondrial carryover is common in plant nuclei preps and is often leveraged to assemble organelle genomes from whole-genome long-read data (Uliano-Silva et al. 2023). Assemblies were annotated with GeSeq (Tillich et al. 2017) using *Viridiplantae* organelle references, followed by a manual check of gene boundaries. Genes encoding tRNAs were predicted with tRNAscan-SE v2.0 (Chan et al. 2021). Final feature tables were exported in GenBank format and circular maps rendered with OGDRAW (Greiner et al. 2019).

## Differential and tissue-enriched gene expression analysis

Raw gene count matrices were generated using StringTie (Pertea et al. 2015) with the prepDE.py script based on transcript assemblies from the 9 tissue types and 2 stress conditions used for RNA-Seq (described in a previous section). These matrices were then used as input for downstream differential expression and tissue-enrichment analyses. Differential expression analysis was conducted in DESeq2 v1.34.0 (Love et al. 2014). For comparative RNA-Seq of leaf tissues from plants grown under drought vs. control and waterlogged vs. control conditions, respectively, an adjusted *P*-value ≤ 0.05 and |log_2_ fold-change|≥ 1 were used as thresholds for the determination of differentially expressed genes (DEGs). For tissue-enriched gene expression analysis, a likelihood ratio test (LRT) was carried out with the full model including tissue type as a factor and the reduced model excluding tissue effects. DEGs with a false discovery rate (FDR) < 0.01 were retained. Normalized counts were obtained using variance-stabilizing transformation, and mean expression was calculated for each tissue. To identify tissue-enriched genes, we assigned each gene to the tissue in which it exhibited the highest expression level, and further required a minimum difference of log_2_ fold-change ≥ 1 between the most and second-most highly expressed tissues. Closely related tissues were collapsed into broader categories (developing leaves + emerging leaves = young leaves; first fully expanded leaves + third fully expanded leaves = mature leaves; root crown + root tips = roots) to increase statistical power and interpretability. Genes were considered expressed if counts per million were ≥ 1 in at least 2 libraries, and stable if their coefficient of variation (CV) across all samples was ≤ 0.20. For all RNA-Seq analyses, gene counts refer to individual alleles of each gene unless stated otherwise.

Both tissue-enriched and stress-responsive DEGs were subjected to functional enrichment analysis. GO term enrichment was conducted using GOATOOLS (Klopfenstein et al. 2018), with significance determined at an FDR-adjusted *p* < 0.05. Summarization and redundancy reduction of enriched GO terms were performed using REVIGO (Supek et al. 2011) with the recommended dispensability cutoff of 0.4. Over-representation was assessed by Fisher’s exact test with Benjamini–Hochberg correction, and visualization was performed as dot plots in R, where dot size reflects the number of DEGs per category and color indicates adjusted significance. For stress-responsive experiments, pathway analysis was carried out in MapMan v3.6 (https://mapman.gabipd.org/) with the AAC Mountainview genome as a reference, and PageMan analysis was conducted using the Ora-Fisher test to determine significance with a cutoff of 1.0. To avoid inflated counts in MapMan analysis, we used OrthoFinder (Emms and Kelly 2019) to group gene models into orthogroups across the 4 haplotypes and then collapsed the 4 alleles to a single representative per orthogroup. A single representative gene ID was then selected for each orthogroup to serve as the identifier in downstream visualization.

For flavonoid pathway analysis (including CT pathway genes), a reference panel of 20 genes was compiled from *Arabidopsis* and *V. vinifera* (Table S1). The full complement of predicted proteins in sainfoin was searched against this panel using DIAMOND BLASTP (Buchfink et al. 2021), and top hits were retained for mapping. The tissue-enrichment status for these candidates was then extracted from the global analysis, and pathway deployment was further summarized by retaining up to 4 top-scoring homolog matches per reference gene. To compare the number of homologs of each of these flavonoid biosynthetic genes (including core CT biosynthesis genes) between AAC Mountainview and the previously published sainfoin genome (He et al. 2024), predicted protein sets from both assemblies were searched using DIAMOND BLASTP (Buchfink et al. 2021) using an E-value cutoff of 1e^−10^, and hits were filtered based on sequence identity (≥ 50%) and alignment coverage (≥ 70% of both query and subject). For each gene family, redundant transcript isoforms were collapsed, and only the top-scoring hit per locus was retained.

## Allele-specific expression analysis

Allele-specific expression patterns under drought and waterlogging stress were investigated by identifying high-confidence allelic gene groups. Predicted protein sequences were assigned to 4 haplotype groups (A, B, C, D) based on chromosome identifiers (e.g., chr1a-chr7a) and analyzed as pseudo-species using OrthoFinder v2.5.4 (Emms and Kelly 2019). Orthogroups containing exactly one gene from each haplotype (1:1:1:1 groups) were selected for further analysis. Orthogroups were filtered to ensure sufficient read support (≥ 50 total reads across haplotypes, ≥ 20 reads per condition, ≥ 10 reads per gene, and at least 2 replicates with detectable levels of expression in each condition). For each allelic group, haplotype-specific expression was presented as the proportion of reads relative to the group’s total expression (i.e., the 4 haplotype proportions sum to 1). Allelic bias within a condition was measured as the difference between the highest- and lowest-expressing haplotypes, with values close to 0 indicating balanced expression and larger values reflecting dominance (with thresholds of 0.25 for mild dominance and 0.5 for strong dominance). Differences in haplotype ratios between conditions (control vs. drought or waterlogging) were tested using chi-square tests in Python (SciPy, statsmodels), with *P*-values adjusted using the Benjamini–Hochberg method (FDR < 0.05). The haplotype contributing the highest proportion in a given condition was defined as the top rank order haplotype, and “rank switching” was recorded when the top rank order haplotype differed between control and stress conditions. We summarized effect sizes as Δ changes in the proportion of the most highly expressed haplotype between conditions (i.e., difference in max-allele proportion). Thresholds of Δ ≥ 0.10 were considered moderate and Δ ≥ 0.20 pronounced. GO term enrichment analyses were conducted specifically on (i) allelic groups showing strong Δ effects (Δ ≥ 0.10 or ≥ 0.20) and (ii) the strong-bias set, following procedures described in the previous section.

## Results

### Assembly of an AAC Mountainview sainfoin reference genome

To generate a high-quality reference genome for a genotype of *O. viciifolia* cultivar AAC Mountainview, we used multiple sequencing platforms and high sequencing coverage to enhance assembly contiguity and resolution. Sequencing of a selected genotype was carried out using short-read Illumina (whole cell), Oxford Nanopore (whole cell), and PacBio HiFi (nuclear) sequencing. Illumina sequencing led to the production of over 700 million read pairs (1.4 billion reads, 210 Gb total), corresponding to 365X coverage of the haploid genome. In the case of Oxford Nanopore sequencing, we obtained 245.6 Gb of long-read data with average read lengths ranging from 7-17 kb and N50 values of 13–36 kb, corresponding to a sequencing depth of 426X. In addition, we also obtained 123.4 Gb of long-read PacBio HiFi data with an average read length of 17.3 kb and an N50 of 17.2 kb, corresponding to 214X coverage (Table S2, Fig. S1). Finally, a total of 439.5 million Hi-C read pairs (132.1 Gb) were generated in the form of Illumina 150 bp paired-end reads (Table S2).

**Fig. 1:**
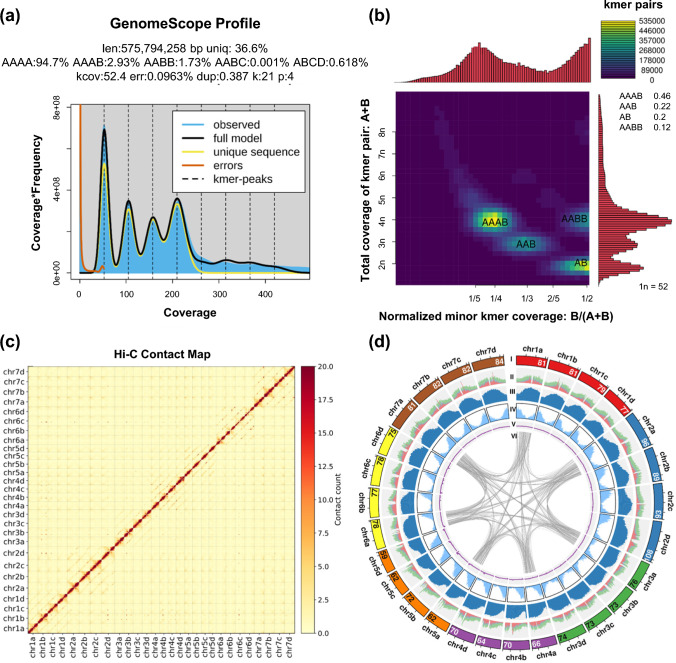
K-mer analysis and genome assembly overview of the sainfoin AAC Mountainview tetraploid genome. **a** K-mer spectrum from GenomeScope analysis. **b** Smudge plot k-mer pair frequency analysis using 21-mers. **c** Hi-C contact matrix of the chromosome-scale genome assembly. The matrix displays chromatin interaction frequencies among the 28 assembled pseudochromosomes, with a strong diagonal with distinct square blocks indicating high-quality chromosome-level scaffolding. Each square along the diagonal represents a single chromosome, with clear separation and limited inter-chromosomal interactions. **d** Circos plot of the final sainfoin pseudochromosome assembly. 28 pseudochromosomes are displayed in a circular layout, grouped by color into 7 chromosome sets (chr1a–chr7d). Six concentric tracks are shown: I. Chromosome ideograms with length (in Mb), color-coded by chromosome group; II. Transposable element (TE) density for Gypsy (red) and Copia (green) elements, shown as stacked histograms; III. CG methylation levels (% mC in CG context) per 1 Mb window; IV. Gene density from annotation; V. GC content profile as a line plot; and VI. Inter-chromosomal synteny links derived from whole-genome alignment, highlighting large-scale structural collinearity between chromosomes groups. For clarity, synteny links are shown for the representative A haplotype only

K-mer analysis with PacBio HiFi reads indicated an estimated haploid genome size of 575.8 Mb and a corresponding tetraploid size of approximately 2.3 Gb (Fig. 1a). Heterozygosity was estimated at 4.7–6.3%, dominated by AAAB (2.9–3.0%) and AABB (1.5–2.0%) k-mer classes. The presence of 4 distinct k-mer coverage peaks and Smudgeplot analysis supported an autotetraploid nature for AAC Mountainview sainfoin (Fig. 1b).

The nuclear genome assembly integrated PacBio HiFi, Nanopore, and Hi-C data, followed by scaffolding and refinement using Hi-C data and polishing using short-read Illumina data. This pipeline yielded a chromosome-level assembly of 2.15 Gb across 28 pseudochromosomes (91.1% of the total 2.36 Gb assembly), each representing 1 of the 4 homologous copies of the 7 base chromosomes with a scaffold N50 exceeding 75 Mb (Fig. 1c, d; Table 1; Table S3). The final assembly exhibited a GC content of 36.2% and individual haplotype assemblies were highly similar in size, ranging from 531.3 Mb (haplotype C) to 547.7 Mb (haplotype D), with an average of approximately 541 Mb, indicating uniform coverage and continuity across haplotypes. Inspection of the Verkko assembly graph showed 7 distinct tangles corresponding to the 7 different chromosome groups and allowed the assignment of unplaced contigs post-scaffolding into chromosomal bins. Hi-C contact maps confirmed the accuracy of the scaffolding, showing strong intra-chromosomal signal and minimal inter-chromosomal interaction (Fig. 1c). BUSCO analysis, which assesses the presence of conserved single-copy orthologs across species, using the embryophyta_odb10 and fabales_odb10 datasets revealed 99.9% and 97.7% completeness, respectively, with 97.2% duplicated genes (Table S4). BUSCO scores for individual haplotypes were comparable, averaging approximately 95–97% completeness, confirming consistent assembly quality across haplotypes (Table S4).

**Table 1:** Summary of AAC Mountainview sainfoin genome assembly metrics

Metric	Value
Total scaffold length (bp)	2,363,917,914
Number of scaffolds	34
Min. number of scaffolds containing half of assembly (L50)	13
Shortest Scaffold from L50 set (N50)	78,148,598
Total contig length (bp)	2,352,489,545
Number of Contigs	3072
Min. Number of Contigs containing half of assembly (L50)	24
Shortest Contig from L50 set (N50)	32,335,739
Number of protein-coding genes	117,890
Number of protein-coding transcripts	143,786
BUSCO completeness (Genome mode, Fabales v10) (%)	97.7%
BUSCO completeness (Protein mode, Fabales v10) (%)	97.7%

To evaluate structural variation, we performed whole-chromosome alignments among the 4 haplotype-resolved copies of each chromosome (e.g., chr1a-chr1d) and between our AAC Mountainview assembly and the cultivar published by He et al. (2024). Haplotype-to-haplotype comparisons in AAC Mountainview demonstrated extensive collinearity, with roughly 85% of aligned sequences being syntenic, supporting the successful phasing of this tetraploid genome (Fig. S2). In inter-cultivar comparisons, where each AAC Mountainview haplotype was aligned to its matching haplotype from the He et al. (2024) study (e.g., chr1a-chr1a), synteny remained high, with 86.9% per chromosome and 82.4% genome-wide when length-weighted. Furthermore, the overall number of structural variants between cultivars was only slightly higher in magnitude than intra-haplotype contrasts (mean 4.22 × 10^4^ vs. 4.07 × 10^4^ structural variants per chromosome, respectively). The most pronounced rearrangements, including inversions, translocations, and small insertions or deletions, were observed on chr5 and chr6, with additional divergence apparent on chr1 and chr2 (Fig. S3). In addition, homolog number comparisons for flavonoid biosynthetic gene families (including those directly involved in CT biosynthesis; Table S1) between AAC Mountainview and the previously published sainfoin genome from an unknown cultivar/accession (He et al. 2024) for the most part indicated similarity between the two assemblies (Table S5). One notable exception was the *CHALCONE ISOMERASE* (*CHI*) family, where a substantially higher number of homologs were identified in AAC Mountainview compared with the previously reported genome (20 vs. 12; Table S5).

## Transposable element landscape and LTR assembly quality

We annotated a total of 2,559,894 repeat elements, comprising over 64% (1.39 Gb) of the 2.15 Gb assembled chromosomal genome (Table 2). LTR retrotransposons were the most abundant TE category, spanning 38.6% of the total masked content with 11.6% Gypsy, 5.4% Copia elements, and 21.7% unclassified LTRs. TIR DNA transposons comprised an additional 12.0% of masked sequence, with Mutator (4.6%), CACTA (3.1%), and hAT (2.5%) elements being the most prominent. Helitrons and long interspersed nuclear elements (LINEs) (primarily L1) accounted for 8.5% and 4.5% of the genome, respectively, while short interspersed nuclear elements (SINEs) and non-LTR retroelements (e.g., pararetrovirus) were minimally represented.

**Table 2:** Summary of transposable element (TE) annotations in the AAC Mountainview sainfoin genome

Class	Subclass	Count	bpMasked	%Masked
LINE	L1	91,409	96,642,763	4.49%
	RTE	3,172	805,260	0.04%
LTR	Copia	153,795	115,201,977	5.35%
	Gypsy	200,132	249,248,137	11.58%
	Unknown	681,387	466,470,968	21.67%
SINE	tRNA	167	50,374	0.00%
TIR	CACTA	128,416	67,056,305	3.12%
	Mutator	350,488	98,411,224	4.57%
	PIF_Harbinger	87,856	27,040,354	1.26%
	Tc1_Mariner	38,858	9,624,527	0.45%
	hAT	163,644	54,818,399	2.55%
nonLTR	Pararetrovirus	1,136	990,143	0.05%
nonTIR	Helitron	569,305	183,610,547	8.53%
Other	Repeat_fragment	90,129	24,094,882	1.12%
Total Interspersed		2,559,894	1,394,065,860	64.77%

*LINE* long interspersed nuclear elements, *LTR* long terminal repeat retrotransposons, *SINE* short interspersed nuclear elements, *TIR* terminal inverted repeat DNA transposons, *nonLTR* non-LTR retrotransposons, *nonTIR* rolling-circle (Helitron) DNA transposons

These extensive intact LTR structures enabled us to evaluate genome contiguity and assembly quality through the LAI. The genome-wide LAI was 14.7 (Table S6), consistent with reference-grade assembly standards (Ou et al. 2018). LAI values of individual chromosomes ranged from 10.2- 24.3, and all 4 haplotypes (A-D) had LAI values above 14.7, exceeding the reference-quality threshold of 10 (Table S6; Fig. S4). Together, these results provide a detailed view of TE architecture and demonstrate assembly contiguity in repeat-rich regions.

## The AAC Mountainview sainfoin genome contains large numbers of rDNA repeats

Multiple copies of rRNA-encoding arrays remain a significant challenge in genome assembly due to their high sequence identity and repetitive nature. We identified a total of 74,779 rDNA features in the AAC Mountainview sainfoin genome. While 20.8% (15,524) were located in the 28 chromosome scaffolds (chr1a–chr7d), the majority were found in chromosome-binned contigs consistent with a lack of divergence between these sequences in the 4 haplotypes (Table S7, Fig. S5). 5S rDNA sequences were primarily identified on chr3 and chr4, while 45S arrays were found on chr5 (Table S7, Fig. S5). In the main chromosomes, rRNA-encoding genes were predominantly on chr3 (5,784), chr4 (5,598) and chr5 (3,993), with 5S loci more abundant than 45S loci. In contrast, unanchored chromosome-associated scaffolds comprised the majority of 45S copies, particularly chr5un (39,487 rDNA annotations, with approximately 13,000 encoding each 45S subunit). Similar tandem arrays were detected on chr3un and chr4un (Table S7). These arrays exhibited tightly linked 5.8S:18S:28S copy ratios, supporting the integrity of the rDNA repeat units. Since nearly all unanchored scaffolds correspond to these rDNA arrays, their organization further supports the overall completeness of the assembly.

## Comprehensive, near-complete gene models with four-allele retention

We integrated an extensive transcriptomic dataset along with homologous protein sequences to construct a comprehensive gene annotation for the AAC Mountainview sainfoin genome. To maximize transcript diversity and improve the detection of condition- and tissue-specific isoforms, Illumina RNA-Seq libraries from 11 distinct tissue types and conditions, spanning vegetative, reproductive, and stress-exposed samples were used as transcript evidence, comprising 44 libraries (4 biological replicates of each) that yielded approximately 2.42 billion strand-specific 150 bp paired-end reads (721.8 Gb). These were complemented by pooled PacBio Iso-Seq full-length transcript reads to further refine exon–intron boundaries and recover long, alternatively spliced transcripts that may be fragmented or missed by short-read data alone. This data consisted of approximately 22.5 million full-length non-chimeric reads and 1.16 million high-quality isoforms, with a mean transcript length of 1.7 kb and a maximum transcript length of 9.6 kb (1.99 Gb).

The final annotation consisted of 117,890 high-confidence protein-coding genes, with an average coding sequence length of 1,297 bp and a mean of 5.3 exons per gene (Table S8). Each haplotype contained approximately 28,700–29,000 genes in total, corresponding to an average of approximately 4,100–4,140 genes per chromosome and supporting an even representation of coding sequences across all 4 haplotypes (Table S8). The gene sets had a very high BUSCO value, exhibiting 97.7% completeness, with less than 0.1% fragmented and 2.3% missing BUSCOs, suggesting a near-complete representation of conserved gene content and affirming the assembly’s high coverage and continuity (Fig. S6). Under a strict criterion (no within-haplotype duplicates), 74.4% of orthogroups were tetra-allelic, 11.4% were tri-allelic, 12.6% were bi-allelic, and 1.7% were mono-allelic (*n* = 21,326 orthogroups). A more lenient analysis that tolerated within-haplotype duplicates gave similar proportions (tetra-allelic: 70.7%, tri-allelic: 13.4%, bi-allelic: 12.9%, mono-allelic: 3.0%; *n* = 27,776). These distributions indicate that the majority of loci retain all 4 alleles in the tetraploid genome.

For functional annotation, we combined sequence homology, domain prediction, and orthology-based inference. In total, between 72.1 and 97.4% of genes matched entries in NCBI NR, TrEMBL, TAIR10, and Swiss-Prot. Pfam domains were assigned to 81.3% of genes, and 90% received Clusters of Orthologous Genes (COG) classifications (Table S9). Overall, more than 97% of predicted genes were annotated by at least one major resource, supporting the completeness and biological relevance of the gene models and providing a complete genomic framework for downstream gene expression, evolutionary, and functional studies in sainfoin. In addition to protein-coding genes, structural annotation also led to the identification of 1,031 putative miRNA precursor loci representing multiple conserved plant miRNA families (Table S10).

## The sainfoin genome contains typical DNA methylation landscapes across genes and transposable elements

**Fig. 2:**
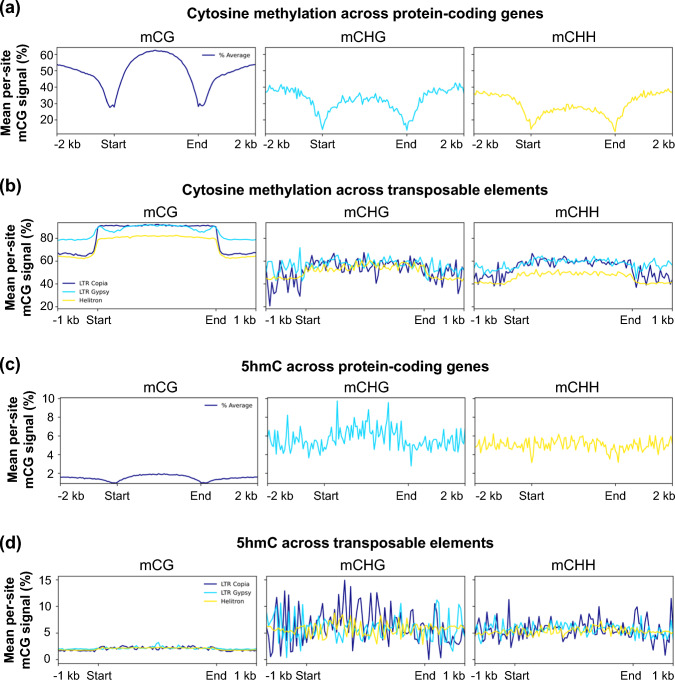
DNA methylation patterns across genes and transposable elements in sainfoin. **a** Mean per-site cytosine methylation (mCG, mCHG, and mCHH) signals across protein-coding genes, including 2 kb upstream and downstream flanking regions. **b** Mean per-site mCG, mCHG, and mCHH signals across three major transposable element (TE) families (LTR Copia, LTR Gypsy, and Helitron), including 1 kb flanking regions. **c** Mean per-site 5-hydroxymethylcytosine (5hmC) signals across protein-coding genes, including 2 kb flanking regions. **d** Mean per-site 5hmC signals across LTR Copia, LTR Gypsy, and Helitron transposable elements, including 1 kb flanking regions. Each line represents the smoothed average across all relevant regions (genes or TE families), aligned by normalized coordinates from start to end. Apparent signal variability in 5hmC and CH contexts likely reflects lower coverage and smoothing effects relative to the denser CG dataset

To characterize the epigenomic landscape of sainfoin, we performed genome-wide DNA methylation profiling using Nanopore data. We observed a typical CG methylation (mCG) profile in genic regions, with low methylation abundance near transcription start and end sites and elevated methylation levels across gene bodies (Fig. 2a). Promoter-proximal regions (2 kb upstream) exhibited low to moderate mCG signal (mean per-site methylation fraction of 45.9%), indicating that most promoters remain in a transcriptionally permissive state rather than being constitutively silenced (Fig. 2a, Fig. S7).

In contrast, TEs showed uniformly high levels of mCG, reflecting strong transcriptional silencing and epigenetic suppression. Family-level analysis revealed distinct patterns: LTR retrotransposons (particularly LTR/Gypsy and LTR/Copia) were densely methylated along their full-length (mean per-site methylation fractions of 89.5% and 91.1%), whereas DNA transposons such as Helitrons showed slightly lower average mCG levels (mean per-site methylation fraction of 81.2%), possibly reflecting variable silencing efficiency or genomic context (Fig. 2b). Notably, pericentromeric regions exhibited the highest mCG levels, which is in line with their heterochromatic structure and transcriptional inactivity (Fig. 1d). Collectively, these results highlight a functionally partitioned epigenome in sainfoin, where gene-rich regions maintain intermediate methylation patterns compatible with expression, while TE-rich heterochromatic domains are heavily methylated to reinforce silencing.

To provide a broader view of cytosine modifications, we also examined non-CG methylation contexts (mCHG and mCHH) and 5hmC. Gene body methylation in mCHG and mCHH signals peaked at 28.9% and 24.2% mean per-site methylation fraction, respectively, but showed a pronounced depletion at transcription start sites, with levels rising outward into both promoter flanks and gene bodies (Fig. 2a; Fig. S7). TE-associated methylation reached mean levels of 55.0–58.8% in the CHG context and 49.0–59.7% in the CHH context (although mCHH was lower in Helitrons specifically, at 49.0%), which was markedly lower than mCG levels (Fig. 2b). Average 5hmC signals across both genes and TEs were uniformly low (typically < 10% of mean per-site fraction) suggesting limited biological prevalence in vegetative tissues or low-confidence detection (Fig. 2c, d; Fig. S7).

## Phylogenetic placement of sainfoin and confirmation of legume-wide whole-genome duplication

To investigate the evolutionary relationship of sainfoin within Fabaceae and interpret its genomic features, we performed comparative genomic analysis using 18 angiosperm species, including 14 legumes and 4 non-legume outgroups. Orthogroup analysis was performed, and a phylogenetic tree constructed from 233 widely shared orthogroups produced a strongly supported topology. *O. viciifolia* was placed within the Hologalegina clade, positioned as a sister group to the *Medicago* lineage alongside other temperate legumes including *Lotus japonicus*, *Pisum sativum*, and *Cicer arietinum* (Fig. S8a).

To better understand the evolutionary dynamics of sainfoin, we also analyzed synonymous substitution rates (K_s_) within the haplotype A genome and among orthologs from selected Fabaceae and non-Fabaceae species to detect whole-genome duplications (WGD). The K_s_ distribution of sainfoin paralogous gene pairs had a distinct peak at K_s_ ~ 0.6 (Fig. S8b), consistent with the legume-wide WGD event previously reported in papilionoid legumes (Cannon et al. 2015). Orthologous K_s_ comparisons between *O. viciifolia* and other legumes showed patterns aligning with expected divergence times. For example, close Fabaceae relatives of sainfoin such as *Medicago truncatula*, *M. sativa*, *C. arietinum*, and *L. japonicus* exhibited K_s_ peaks near 0.4–0.5, suggesting divergence occurred after the legume WGD. More distantly related legumes (e.g., *Arachis hypogaea*), on the other hand, displayed slightly higher K_s_ peaks (approximately 0.55), which suggests a somewhat earlier divergence. In contrast, non-legume outgroup species exhibited wide, high K_s_ peaks (> 1.25), indicative of a more ancient divergence and past polyploidy events (Fig. S8b). Together, these findings demonstrate that sainfoin retains clear molecular signatures of both ancient and group-specific WGD events.

## Complete assembly and annotation of sainfoin organelle genomes

In addition to the nuclear assembly, we also assembled and circularized chloroplast and mitochondrial genomes. The chloroplast genome assembled as a single circular molecule of 122,397 bp (GC = 34.6%), with 78 protein-coding genes, 24 tRNA-encoding genes, and 4 rRNA-encoding genes (Table S11; Fig. S9). These included photosystem-related *PSA* and *PSB* genes, as well as genes putatively encoding ribulose-1,5-bisphosphate carboxylase/oxygenase large subunit (rbcL), ATP synthase (atpA-H), RNA polymerases (rpoA-C), ribosomal protein large/small subunits (RPL/RPS), NADH dehydrogenase subunits, and cytochrome b6/f subunits (petB and petD; Table S11).

The mitochondrial genome was assembled as a single 321,160 bp contig (GC = 45.2%). We annotated 50 genes in total, including 31 conserved protein-coding genes, 16 tRNA-encoding genes, and 3 mitochondrial rRNA-encoding genes (*rrn*5, *rns*, *rnl*), encompassing the core angiosperm mitochondrial gene complement (Table S12; Fig. S10). The protein-coding genes included those putatively encoding ATP synthase subunits (atp1/4/6/8/9), cytochrome b (cob), cytochrome-c oxidase subunits (cox1/2/3), NADH dehydrogenase (complex I) subunits (nad1/3/4/4L/5/6/7/9), cytochrome-c maturation factors (ccmB/C/FC/FN), the twin-arginine translocase component (tatC), the group II intron maturase (matR), and ribosomal proteins (rpl5, rpl16, rps1/3/4/12/14). Numerous additional hypothetical open reading frames (ORFs; ≥ 100 aa) were also present (Table S12), often in multiple copies, consistent with the repeat-rich nature of plant mitogenomes.

## Tissue-enriched gene expression patterns reveal functional specialization

**Fig. 3:**
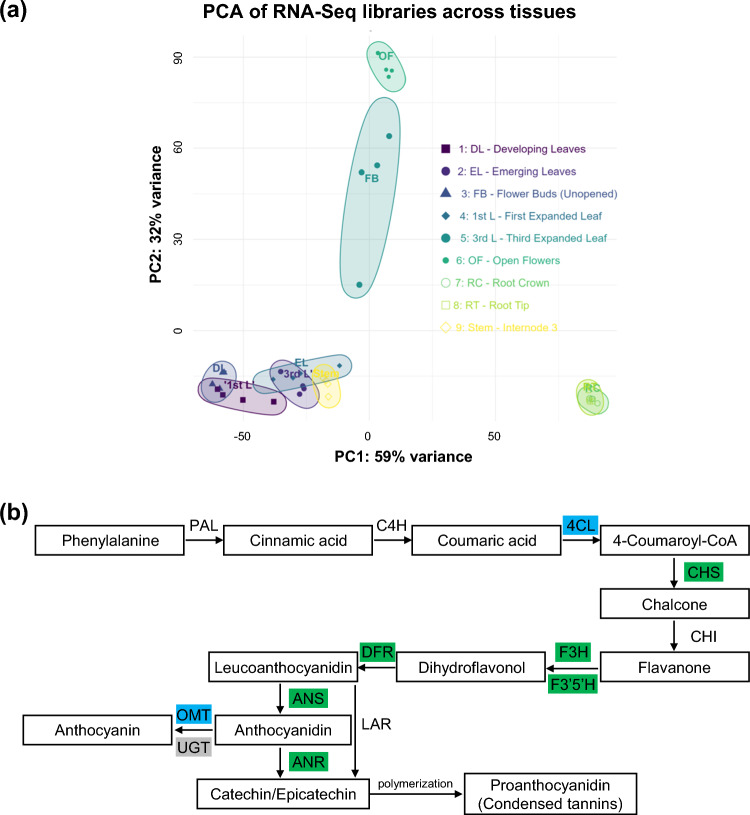
Transcriptome sampling and tissue-specific gene expression patterns. **a** Principal component analysis (PCA) of RNA-Seq libraries from 9 tissues. Samples cluster by tissue type, with clear separation between root (RT, RC), flower (FB, OF), stem, leaves (DL, EL, 1st L, and 3rd L). **b** Flavonoid to condensed tannin biosynthetic pathway with enriched enzymes highlighted. Enzymes encoded by genes identified as tissue-enriched in sainfoin are highlighted in green (young leaves), blue (stem), and grey (roots). *PAL* phenylalanine ammonia-lyase, *C4H* cinnamate-4-hydroxylase, *4CL* 4-coumarate:CoA ligase, *CHS* chalcone synthase, *CHI* chalcone isomerase, *F3H* flavanone 3-hydroxylase, *F3′5’H* flavonoid 3’, 5’-hydroxylase, *DFR* dihydroflavonol reductase, *ANS* anthocyanidin synthase, *ANR* anthocyanidin reductase, *LAR* leucoanthocyanidin reductase, *OMT* O-methyltransferase, *UGT* UDP-glycosyltransferase

To examine gene expression patterns associated with tissue identity and function, we performed tissue-enriched gene analysis using RNA-Seq data from 9 sainfoin tissues representing key developmental stages (Fig. 3a). We identified 86,084 expressed genes (counts per million were ≥ 1 in at least 2 libraries), of which 33,989 were expressed across all developmental stages and tissues assessed (Data S1). Among these ubiquitously expressed genes, 219 were stably expressed across all tissues with a CV ≤ 0.20 (Data S1). Applying a more stringent cutoff (CV < 0.15) reduced this set to only 5 genes (annotated as ankyrin repeat proteins and a DEAD-box RNA helicase), and none met the CV < 0.10 threshold (Data S1), underscoring the difficulty of identifying universally invariant transcripts in a multi-tissue dataset.

To assess tissue bias, genes with an FDR < 0.01 and a log_2_ fold-change difference of at least 1 between the highest and second highest expressing tissues were retained, resulting in high-confidence tissue-specific gene sets (Fig. S11, Data S2). In total, our tissue-enrichment pipeline identified 12,586 genes with clear organ-biased expression. GO enrichment analysis of the tissue-enriched gene sets confirmed their biologically coherent expression patterns (Data S2). For example, flower buds showed strong enrichment for genes involved in pollen development, cytoskeletal dynamics, and energy metabolism. In root tissues, genes linked to ion transport, stress signaling, and xenobiotic detoxification were enriched. Stem tissues showed high expression of genes involved in secondary cell wall biosynthesis, particularly lignin and xylan pathways. Genes associated with photosynthesis, plastid function, and pigment biosynthesis were enriched in young leaves, while nutrient transport, senescence, and salicylic acid-mediated response-related genes were up-regulated in mature leaves (Data S2).

Within this framework, we specifically examined genes in the flavonoid biosynthetic pathway, including a core set of genes involved in CT biosynthesis. Among 20 curated candidates mapped from *Arabidopsis* and *V. vinifera* references (Table S1), 9 were tissue-enriched in our atlas (Fig. 3b). Six were biased toward young leaves, including those putatively encoding anthocyanidin synthase (ANS; also known as leucoanthocyanidin dioxygenase [LDOX]), chalcone synthase (CHS), anthocyanidin reductase (ANR), flavanone 3-hydroxylase (F3H), flavonoid 3’, 5’-hydroxylase (F3′5’H), and dihydroflavonol reductase (DFR). The remaining 3 were enriched in root (UDP-glycosyltransferase [UGT]) and stem (O-methyltransferase [OMT] and 4-coumarate coenzyme A ligase [4CL]; Fig. 3b).

## Shared and distinct transcriptional responses to contrasting water stresses

**Fig. 4:**
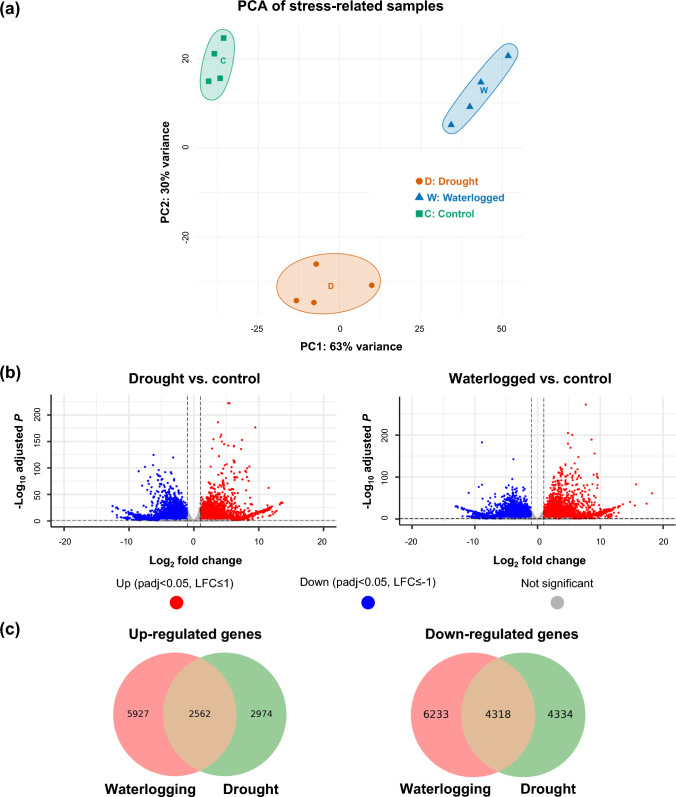
Transcriptomic responses to drought and waterlogging stress in sainfoin. **a** Principal component analysis (PCA) of RNA-Seq samples from leaves derived from control (C), drought (D), and waterlogged (W) plants. **b** Volcano plots showing differentially expressed genes (DEGs) between drought vs. control (left) and waterlogging vs. control (right). Genes with log₂ fold-change > 1 and adjusted *P*-value < 0.05 are highlighted in red. **c** Venn diagrams showing overlap of up-regulated and down-regulated DEGs between drought and waterlogging treatments. Substantial overlap suggests a shared transcriptional response to both types of water stress

To examine the transcriptomic response of sainfoin to drought and waterlogging, we also carried out RNA-Seq using leaf tissues (third from the shoot tip) from plants subjected to 7 days of drought and waterlogging, respectively, and compared to leaves of the same stage grown under normally watered conditions (Fig. 4a). Following drought stress, 14,188 gene models (including all alleles of each gene) were differentially expressed compared to the normally watered control, including 5,536 that were up-regulated and 8,652 that were down-regulated. Similarly, we observed 19,040 DEGs following waterlogging compared to the normally watered control, with 8,489 being up-regulated and 10,551 being down-regulated (Fig. 4b). Many DEGs exhibited large log_2_ fold-changes (> 10) and highly significant adjusted *P*-values, suggesting strong transcriptional responses to stress (Fig. 4c; Data S3). Interestingly, although drought and waterlogging represent contrasting stress conditions, a considerable number of DEGs were shared between them, with 2,562 common gene models up-regulated and 4,318 down-regulated under both treatments (Fig. 4c), indicating a conserved core response to water-related stress.

GO term enrichment analysis of DEGs between drought and control conditions, as well as waterlogging and control conditions, indicated that several terms were common to both types of stress (Fig. S12, Data S4). For up-regulated DEGs, leaves from both drought and waterlogged plants exhibited enrichment in terms such as reactive oxygen species (ROS) metabolic process, regulation of hydrogen peroxide biosynthetic process, and oxidoreductase activity, for example. In the case of down-regulated DEGs, common GO terms that were significantly enriched included photosynthesis and signaling receptor activity (Fig. S12, Data S4).

However, many significantly enriched GO terms were specific to each type of stress. For example, up-regulated DEGs from the drought experiment showed significant GO term enrichment in response to water deprivation, photosystem II (PSII) repair, regulation of cutin biosynthetic process, phenylpropanoid biosynthetic process, carotene biosynthetic process, and antioxidant activity. Furthermore, several GO terms related to plant cell walls were also enriched, including cell wall organization or biogenesis, plant-type secondary cell wall biogenesis, regulation of secondary cell wall biogenesis, and plant-type cell wall. Drought-specific enriched GO terms for down-regulated DEGs, on the other hand, included light harvesting in PSII, flavonoid biosynthetic process, regulation of stomatal opening, and calcium ion binding, for example (Fig. S12, Data S4). In the case of waterlogging-specific enriched GO terms for up-regulated DEGs, these included response to abiotic stimulus, regulation of abscisic acid (ABA)-activated signaling pathway, hormone metabolic process, and superoxide anion generation. For down-regulated DEGs, waterlogging-specific enriched GO terms included many terms related to photosynthesis, such as photosynthesis light reaction, carbon fixation, fructose 6-phosphate metabolic process, regulation of photosynthesis light reaction, regulation of photorespiration, regulation of photosynthesis, transferring electrons within the cyclic electron transport pathway of photosynthesis activity, and fructose 1,6-bisphosphate-1-phosphatase activity. In addition, several terms related to ribosomes, including ribosome biogenesis, rRNA transport, and structural constituent of ribosome, were also significantly enriched. Finally, GO terms such as generation of precursor metabolites and energy, as well as cell wall organization or biogenesis, were also significantly enriched specifically in DEGs that were down-regulated under waterlogging (Fig. S12, Data S4).

**Fig. 5:**
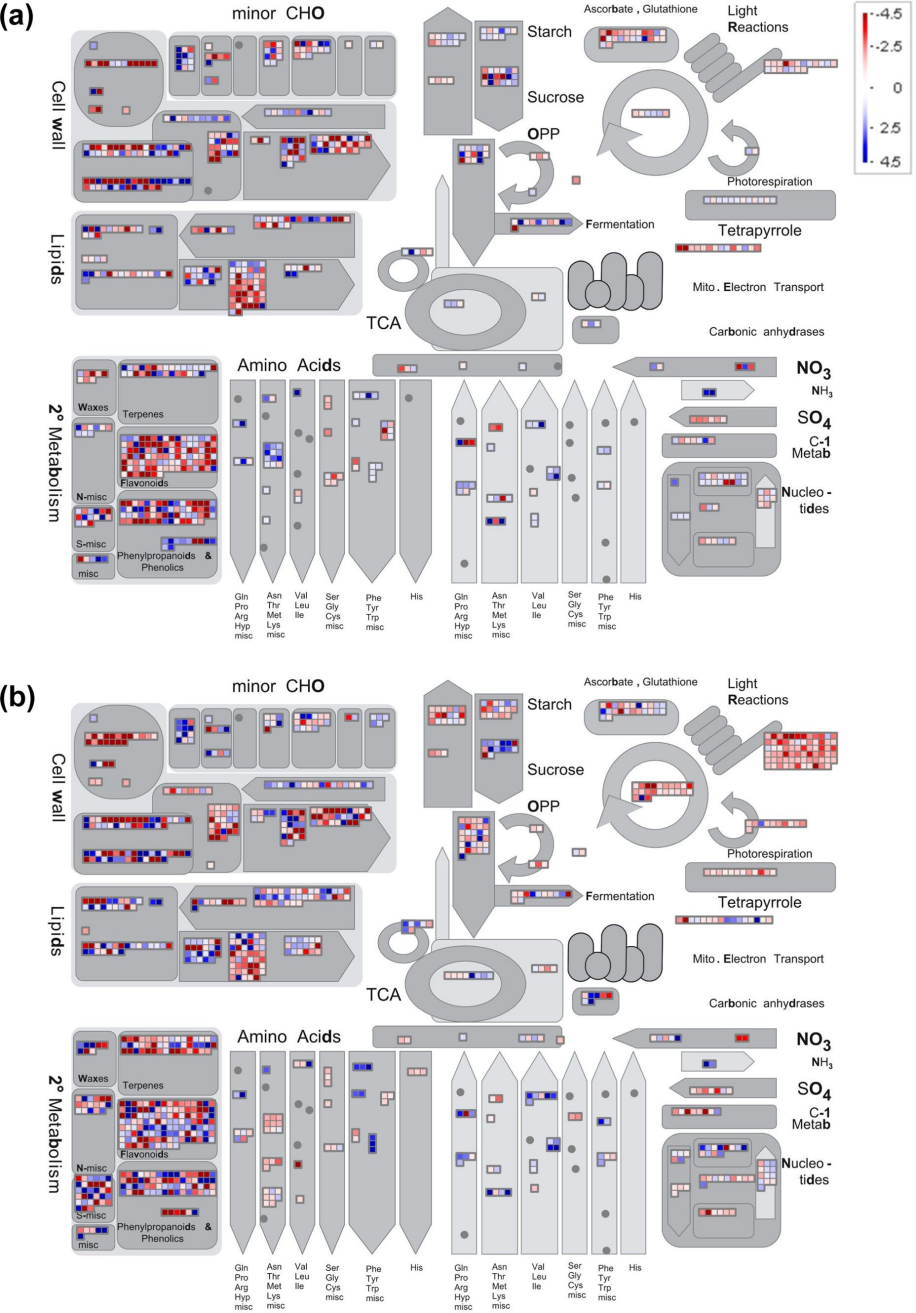
Transcriptional responses in general metabolic pathways of sainfoin leaves to drought and waterlogging stress. Differentially expressed genes (DEGs; FDR ≤ 0.05) between drought and well-watered conditions (**a**) and between waterlogged and normally watered conditions (**b**). Red/pink boxes denote down-regulated DEGs, while blue boxes indicate up-regulated genes. Gray boxes/shapes represent different pathway groupings according to the MapMan program, and small circles indicate that no DEGs were observed in a particular pathway. *CHO* carbohydrates, *OPP* oxidative pentose phosphate pathway, *TCA* tricarboxylic acid cycle

To complement GO term analysis, we also carried out PageMan and MapMan pathway analysis to further unravel shared and distinct global transcriptional reprogramming under drought and waterlogged conditions. Both drought and waterlogging induced similar responses in pathways/families related to ABA, abiotic stress, major carbohydrate degradation, cell wall precursor synthesis, chromatin structure, and AP2/EREBP transcription factors (up-regulated genes over-represented), as well as auxin, ethylene, and receptor kinase signaling (down-regulated genes over-represented) (Fig. 5a, b; Fig. S13; Fig. S14; Data S5). However, as was the case with GO term analysis, we also observed considerable stress-specificity in certain pathways/categories. For example, drought elicited the over-representation of up-regulated DEGs involved in peroxiredoxin, dismutases and catalases, hemicellulose synthesis, and prokaryotic ribosomal protein synthesis, as well as the over-representation of down-regulated DEGs related to cytokinin, transport (particularly nutrients), calcium and MAP kinase signaling, WRKY transcription factors, and ubiquitin (Fig. 5a; Fig. S13; Fig. S14; Data S5). Conversely, DEGs that were up-regulated under waterlogging were over-represented in pathways/categories related to salicylic acid, transport (particularly ions and amino acids), DOF zinc finger transcription factors, homeobox transcription factors, MYB domain transcription factors, bZIP transcription factors, and ubiquitin, for example. In terms of down-regulated genes, waterlogging elicited far more substantial transcriptional changes than drought in almost all categories related to photosynthesis, with down-regulated genes being largely over-represented. Similarly, waterlogging also elicited over-representation of down-regulated DEGs involved in cellulose synthesis, cell wall degradation, eukaryotic ribosomal protein synthesis, and ribosome biogenesis (Fig. 5b; Fig. S13; Fig. S14; Data S5).

## Allele-specific expression and haplotype bias

**Fig. 6:**
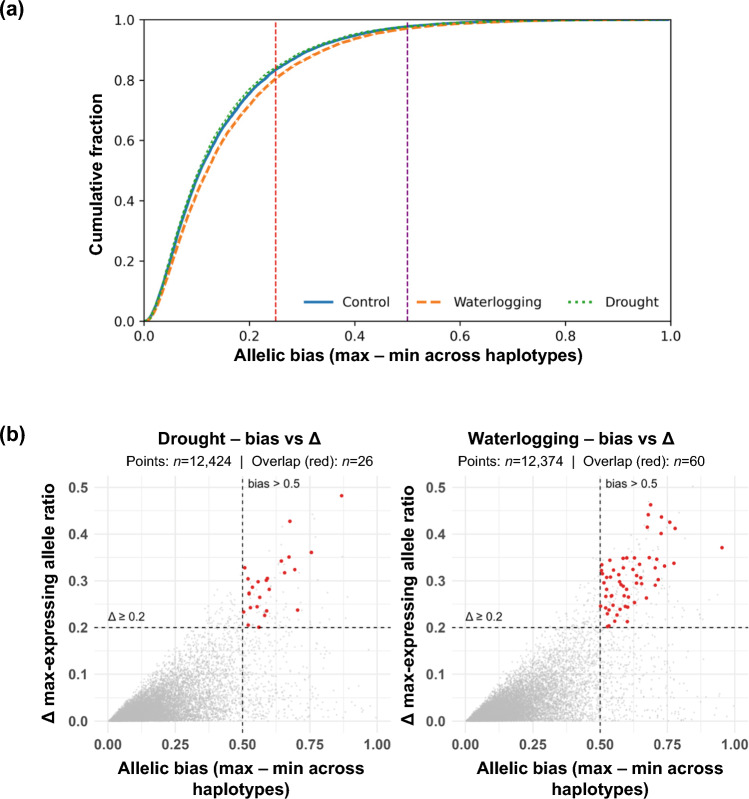
Allele-specific expression bias distributions in sainfoin under control and stress conditions. **a** Cumulative distribution functions of allele expression bias (max–min haplotype ratio) across 1:1:1:1 orthogroups are shown for control (blue), waterlogging (orange), and drought (green) conditions. Red dashed lines indicate mild dominance threshold (0.25). Purple dashed lines indicate strong dominance threshold (0.5). **b** Scatter plots show allelic bias (x-axis) versus change in the top-allele ratio between stress and control (Δ, y-axis). Dashed lines indicate thresholds (bias > 0.5, Δ ≥ 0.2). Red points denote quartets that are both strongly biased and undergo rank-order switching (Δ ≥ 0.2), while gray points indicate all others

To examine allele expression bias in tetraploid sainfoin, we performed allele-specific expression analysis using our haplotype-resolved gene models and RNA-Seq data from leaf tissues derived from plants grown under control and stress (drought and waterlogging) conditions. Among 12,424 high-confidence homologous gene groups representing all 4 haplotypes (A–D), most exhibited mild expression bias, with the distribution skewed toward lower bias values (bias < 0.25, defined as the difference between the highest and lowest expressed homolog), consistent with broadly balanced haplotype-level expression (Fig. 6a). Only 2.6–3.0% of quartets (320–370 gene models) showed strong expression dominance (bias > 0.5, indicating functional dominance of a single allele), suggesting that hardwired *cis*-regulatory dominance is relatively uncommon in sainfoin.

To assess regulatory dynamics under stress, we examined rank-order switching events where the top-expressing homolog changed between control and stress conditions. Strikingly, more than one-third of gene quartets (4,435 under drought, 4,774 under waterlogging) exhibited such switching, indicating widespread but subtle regulatory shifts. In most cases, these changes were modest. Indeed, 93.2% of drought-related and 91.2% of waterlogging-related rank-switched gene quartets had a delta max expression ratio < 0.1 (i.e., the difference in maximum-expressing allele ratios between stress and control), suggesting fine-scale tuning rather than major allelic shifts. After applying explicit effect-size filters, only a small subset remained (waterlogging: 420 quartets at Δ ≥ 0.1 and 104 at Δ ≥ 0.2; drought: 302 at Δ ≥ 0.1 and 61 at Δ ≥ 0.2). Notably, the overlap between genes with strong expression bias and those undergoing dominance switching remained limited (60 and 26 genes per condition Δ ≥ 0.2), implying that highly biased alleles are generally insulated from environmental perturbation (Fig. 6b). GO term analysis was consistent with this view: rank-switching sets (Δ ≥ 0.1/0.2) showed no enriched terms (FDR < 0.05) under either stress, whereas strong-bias sets yielded a handful of stress-relevant categories, including monooxygenase activity, secondary metabolite biosynthetic process, and oxidoreductase activity in the case of the drought vs. control comparison, and cell-wall modification in the case of the waterlogging vs. control comparison (Table S13). Together, these findings suggest that sainfoin employs a flexible yet balanced regulatory system, where most genes operate under mild haplotype bias and stress elicits fine-scale tuning rather than widespread dominance-like behavior, reinforcing the general stability of the expression landscape.

## Discussion

Despite the advantages of sainfoin as a forage crop, its improvement has been relatively slow compared to other forages, at least in part due to the paucity of associated genomic resources. While an unknown cultivar of ‘common’ sainfoin was recently sequenced (He et al. 2024), most modern sainfoin cultivars contain both ‘common’ and ‘giant’ type backgrounds (Mora-Ortiz and Smith 2018), and we thus aimed to generate a comprehensive genome and transcriptome for a more representative cultivar of sainfoin to further support both basic biology and practical breeding applications. To achieve this, we sequenced a single genotype of the AAC Mountainview cultivar using PacBio and Oxford Nanopore long-read sequencing, Illumina short-read sequencing, and Hi-C data for scaffolding, generating a highly contiguous and complete 2.36 Gb genome that was resolved into 28 pseudochromosomes representing 4 homologs of the 7 base chromosomes (Fig. 1, Table 1).

Whole-genome alignments among the 4 haplotypes showed extensive collinearity and few structural rearrangements (Fig. S2), suggesting a genetically balanced nature. Interestingly, the AAC Mountainview genome is considerably larger than the previously published 1.95 Gb assembly of an undisclosed Chinese sainfoin cultivar (He et al. 2024), and our assembly also displayed some other notable differences, particularly on chromosomes 2, 5, and 6, including indels, translocations, and inversions (Fig. S3). These disparities could be due to cultivar-specific variation, repeat handling strategies, and/or differences in assembly methodology. Phylogenomic analyses placed sainfoin within the Hologalegina clade and suggested that it underwent the papilionoid-wide WGD that occurred approximately 58 million years ago, which is consistent with previous studies (He et al. 2024; Fig. S8). Our findings align with genome evolution across other Fabaceae species, where extensive chromosomal synteny is generally preserved following the papilionoid-wide WGD (Cannon et al. 2015).

Repeat analysis indicated that over 64% of the genome consists of repetitive elements, with the largest proportion comprising LTR retrotransposons (Table 2), which corresponds with previous findings in sainfoin (He et al. 2024), as well as other autopolyploid temperate legumes such as alfalfa (Shen et al. 2020). Among these, Gypsy (11.6%) and Copia (5.4%) elements predominate in sainfoin, with 21.7% of the genome being composed of unclassified LTRs (Table 2), likely reflecting highly diverged or lineage-specific retrotransposons not captured in existing repeat libraries. Furthermore, the current sainfoin genome assembly possessed an LAI of 14.7, with individual chromosome scores ranging from 10.7 to 24.3 and all 4 haplotypes individually exceeding the reference-quality threshold of 10 (Ou et al. 2018; Fig. S4; Table S6). This measure of genome continuity was substantially higher than the recently published ‘common’ sainfoin genome, which exhibited LAI scores between 4.8 and 7.1 per haplotype (He et al. 2024). This could possibly reflect the advantage of including long-read PacBio HiFi sequencing for recovering intact retrotransposons, illustrating the impact of sequencing technology on TE representation (Pucker et al. 2022).

An unexpected yet biologically intriguing outcome of our sainfoin genome assembly was the identification of an extraordinarily large number of high-copy tandem rRNA-encoding gene arrays on unanchored chromosome-associated scaffolds (Fig. S5; Table S7). Even with extensive long-read data and Hi-C scaffolding, most 45S rDNA sequences could not be anchored, possibly due to the fact that 45S rDNA loci are typically located in terminal chromosomal regions (Roa and Guerra 2012). Despite being unanchored, the structure and copy number of these arrays, especially the approximately 13,000 copy units per subunit on chr5un amounting to nearly 40,000 rDNA units with a balanced 5.8S:18S:28S ratio, make it likely that they are functional nucleolar organizer regions (NORs) rather than fragmented assembly artifacts. While rDNA copy number can vary substantially across plant species/accessions (Hasterok et al. 2006), even high-quality long-read assemblies frequently break at rDNA arrays (Igolkina et al. 2025), underscoring both their biological scale and the persistent technical difficulty of resolving them. While the majority of other legumes previously surveyed have primarily been assessed at the cytogenetic level for rDNA organization (e.g., Abirached-Darmency et al. 2005), unusually large arrays are known to influence regulation in other plants, including nucleolar dominance in *Brachypodium hybridum* (Borowska-Zuchowska et al. 2021) and NOR activity in wheat (Tulpová et al. 2022). The extensive arrays observed in sainfoin therefore establish a valuable reference point for investigating rDNA organization and potential regulatory effects in forage legumes.

Gene annotation yielded 117,890 high-confidence protein-coding genes (Table 1), with each haplotype bearing approximately 28,700–29,000 genes in total, supported by a BUSCO score of 97.7% (Fabales) and over 97% of genes having been successfully annotated across multiple databases (Table S4; Fig. S6). For comparison, recent assemblies reported 44,623 haploid equivalent genes in *M. truncatula* (Pecrix et al. 2018), 47,526 in *P. sativum* (Yang et al. 2022), 28,251 in *L. japonicus* (Li et al. 2020) and approximately 41,000 in *M. sativa* (Chen et al. 2020). In comparison, 109,998 high-confidence genes, with an average of 27,284 genes per haplotype, were identified in the previous ‘common’ tetraploid sainfoin assembly (He et al. 2024). This difference between sainfoin genomes could reflect biological factors and/or discrepancies in the technical aspects of annotation, with the annotation pipelines used in our study potentially enabling broader capture of the gene space. Furthermore, the incorporation of long-read Iso-Seq data proved essential to our annotation process, as it allowed for the recovery of nearly 10,000 transcripts and gene isoforms that short-read RNA-Seq evidence alone had missed. In addition to protein-coding genes, the AAC Mountainview sainfoin genome also contained 1,031 putative miRNA precursor loci representing multiple conserved plant miRNA families (Table S10; Guo et al. 2020), providing a useful resource for future comparative and functional analyses.

In plants, cytosine methylation occurs in three sequence contexts, including CG, CHG, and CHH (H = A, T, or C), and underpins genome stability, TE silencing, and gene regulation. Among these, mCG is typically the most abundant and stably maintained mark in somatic tissues (Law and Jacobsen 2010). In sainfoin, we found pericentromeric regions to display the highest mCG levels (Fig. 1d), corresponding to their heterochromatic nature (Meyer 2010). Furthermore, gene body mCG was enriched (with only modest levels of non-CG methylation) (Fig. 2a; Fig. S7), which could play putative roles in expression stabilization, splicing accuracy, protection against TE insertions and/or minimization of aberrant transcription, although the precise role of this type of methylation remains controversial (Muyle et al. 2022). Promoter-proximal regions (2 kb upstream of transcriptional start sites) exhibited lower levels of mCG (Fig. 2a), which is consistent with a permissive chromatin state that typically facilitates transcription initiation (Qiao et al. 2025). In contrast, TEs, and especially LTR retrotransposons, displayed dense mCG, mCHG and mCHH (Fig. 2b), suggesting strong epigenetic silencing (Liu and Zhao 2023). In comparison, DNA transposons such as Helitrons showed slightly reduced mCG levels, which could indicate differential regulation or less recent transpositional activity (e.g., Vonholdt et al. 2012). Similar patterns have been observed in soybean, where mCG predominates in gene bodies, while non-CG methylation (particularly mCHH) is enriched in TE and heterochromatic regions (e.g., Song et al. 2013). Beyond mCG, mCHG, and mCHH, we also examined the occurrence of 5hmC, which is an oxidative form of cytosine methylation; the presence and biological functions of which are not yet entirely clear in plants (e.g., Erdmann et al. 2014; Wang et al. 2015b). As expected, levels of 5hmC were low across the sainfoin genome (< 10% of mean per-site fraction; Fig. 2c, d; Fig. S7), suggesting limited prevalence in young leaves, which is consistent with previous reports in other plant species (e.g., Wang et al. 2015b).

In addition to the nuclear genome, we also assembled complete chloroplast and mitochondrial genomes for AAC Mountainview sainfoin. The 122.4 kb chloroplast genome lacks the large inverted repeat (IR) and therefore does not exhibit the quadripartite large single-copy (LSC)-IR-small single-copy (SSC)-IR that is typical in many plant species (Wicke et al. 2011; Fig. S9). This structure is instead consistent with legumes of the IR-lacking clade (IRLC) and with *Onobrychis* plastomes in particular (Moghaddam et al. 2022). It was found to contain 78 protein-coding genes, 24 tRNA-encoding genes, and 4 rRNA-encoding genes, as well as a GC content of 34.6%, which correlates well with previous findings in sainfoin (Jin et al. 2021). This includes the full complement of genes for PSI and PSII, RubisCO, ATP synthase, ribosomal proteins, RNA polymerase subunits, and the NADH dehydrogenase complex (Table S11), all with expected intron structures typical of angiosperm plastomes (Daniell et al. 2016).

The mitochondrial genome, on the other hand, was resolved as a 321.2 kb contig (Fig. S10), which is intermediate in size compared to other Papilionoideae legumes with sequenced mitochondrial genomes (Choi et al. 2019). The genome possessed a GC content of 45.2% and contained 50 genes in total, encompassing 31 protein-coding genes, as well as genes encoding 16 tRNAs, 3 rRNAs and numerous hypothetical ORFs (≥ 100 aa), often in multiple copies, consistent with the repeat-rich nature of plant mitogenomes (Wynn and Christensen 2019). Protein-coding genes included those coding for respiratory chain complexes and ribosomal proteins (Table S12), which is similar to observations in other members of Fabaceae (Choi et al. 2019). To the best of our knowledge, this is the first mitochondrial genome to be characterized for sainfoin, providing additional information for downstream genomic analyses in this species.

In addition to genomic resources, we also generated a large volume of transcriptomic data from 9 different developmental stages/tissue-types (Fig. 3a), which provides biological context for downstream assays. The small set of genes that were expressed in a relatively stable manner across tissues (219 at CV ≤ 0.20; 5 at CV < 0.15; none at CV < 0.10) offers practical qRT-PCR reference genes (Data S1), but the sharp drop with stricter thresholds cautions that universal housekeeping controls are rare across diverse plant tissues. This suggests that multi-gene normalization may be needed under specific experimental designs. In parallel, our tissue-enrichment pipeline identified 12,586 genes with clear organ-biased expression with strong concordance between functional enrichment and the tissue of highest expression (Fig. S11; Data S2), such as pollen development and cytoskeletal remodeling in flower buds, ion transport and stress signaling in roots, lignin/xylan biosynthesis in stems, and photosynthesis and pigment metabolism in young leaves (Data S2).

Within this framework, we also found that the majority of core CT biosynthetic genes exhibited leaf-biased expression, including those encoding CHS, F3H, F3′5’H, DFR, ANS, and ANR (Fig. 3b). This distribution aligns with the well-established preferential accumulation of CTs in young leaves of sainfoin (Li et al. 2014) and their suggested functions in the protection against insect pests, oxidative stress, and plant pathogens (Dixon and Sarnala 2020). Interestingly, two genes that are involved in the conversion of anthocyanidins to anthocyanins rather than CTs (*OMT* and *UGT*) instead exhibited stem- and root-biased expression, respectively (Fig. 3b), which could also contribute to the preferential channeling of precursors into CT production in sainfoin leaves. In order to place these findings in a genomic context, we also compared the copy numbers of flavonoid biosynthetic gene families (including core CT biosynthesis genes) between AAC Mountainview and the previously published sainfoin genome (He et al. 2024). The vast majority of these families showed broadly similar numbers of putative homologs between the two assemblies (Table S5), indicating that the flavonoid biosynthetic gene complement is largely conserved between these genomes. Minor differences in gene copy number likely reflect variation in gene annotation, assembly resolution, or small-scale duplication and loss events commonly affecting plant gene families (Panchy et al. 2016). The *CHI* family, on the other hand, included 20 homologs in the AAC Mountainview genome and only 12 homologs in the previously sequenced sainfoin genome (He et al. 2024; Table S5), which could reflect a higher level of copy number variation between the two genotypes. However, further study will be required to determine the biological significance of this finding. Taken together, these results confirm the CT pathway expansion detected previously in sainfoin (He et al. 2024) while adding cross-tissue resolution.

Since sainfoin’s agronomic performance is constrained by limited resilience to abiotic stresses such as drought and waterlogging (e.g., Heinrichs 1970; Biligetu et al. 2014), and improving tolerance to these environmental challenges remains a key breeding target, we also carried out RNA-Seq with sainfoin leaves derived from plants subjected to drought, waterlogging, and normally watered conditions (Fig. 4a, b). Sainfoin leaves exhibited a notable overlap in transcriptional responses to drought and waterlogging, with more than 6,800 gene models (alleles) common to both conditions (Fig. 4c; Data S3). Overall, sainfoin underwent a shift in ABA-related signaling, along with an increase in the expression of genes related to ROS biosynthesis, carbohydrate degradation, cell wall precursors, and the production of AP2/EREBP transcription factors, as well as a decrease in the expression of genes related to photosynthesis, receptor kinase signaling, and auxin/ethylene pathways (Fig. 5a, b; Fig. S12; Fig. S13; Fig. S14; Data S4; Data S5).

These changes were overlaid by an even larger subset of DEGs that were specific to either drought or waterlogging stress (Fig. 4c; Data S3). In the case of drought, this included an enrichment of up-regulated genes involved in cell wall reinforcement, the production of cutin, and increases in antioxidants and/or their activities, as well as an enrichment in down-regulated genes involved in stomatal opening, flavonoid biosynthesis, nutrient transport, calcium- and MAP kinase signaling, and WRKY transcription factors (Fig. 5a; Fig. S12; Fig. S13; Fig. S14; Data S4; Data S5). Waterlogging, on the other hand, led to a general up-regulation in genes involved in the salicylic acid pathway, the transport of ions and amino acids, and many DOF, HB, MYB, and bZIP transcription factors, as well as declines in the expression of genes involved in energy production, cell wall metabolism, and ribosome biogenesis (Fig. 5b; Fig. S12; Fig. S13; Fig. S14; Data S4; Data S5). While many of these changes align with common transcriptional responses that benefit plants under drought and hypoxia resulting from waterlogging (e.g., Mukarram et al. 2021; Pan et al. 2021), others, such as decreases in signaling (e.g., Schulz et al. 2021), flavonoid biosynthesis (e.g., Baozhu et al. 2022), energy-related pathways (e.g., Zahra et al. 2021), and ribosome production (Dias-Fields and Adamala 2022), as well as a potentially excessive accumulation of ROS (e.g., Fujita and Hasanuzzaman 2022), provide potential targets for the future improvement of abiotic stress tolerance in sainfoin. Although these findings yield a molecular framework for future breeding endeavors aiming to improve water-related stress tolerance in sainfoin, the integration of shoot and root transcriptomes at various time points, small RNA profiling, metabolomic data, and plant growth/morphology changes will be essential to connect molecular states to the physiological adaptations that determine sainfoin performance under variable water regimes.

Sainfoin is largely considered to be an autotetraploid (He et al. 2024); however, this has been debated and it has also been suggested that it could instead be an allotetraploid derived from very closely related progenitors (Özturk and Tek 2024). While allopolyploid genomes can display strong subgenome dominance and homoeolog expression bias, this is generally not observed in autopolyploids (Alger and Edger 2020). In line with an autotetraploid interpretation for sainfoin (homologous haplotypes rather than divergent subgenomes), our allele-specific expression analysis showed that sainfoin retains a relatively balanced expression profile, with the majority of homologous gene quartets showing only mild to moderate expression bias that was largely stable across normally watered, drought and waterlogging stresses (Fig. 6). However, a small proportion of alleles exhibited strong expression bias (approximately 3–4% of alleles) across control and stress conditions (Fig. 6), with biologically plausible enrichment in GO terms including monooxygenase/oxidoreductase activity and secondary metabolite biosynthesis under drought, and cell wall modification under waterlogging (Table S13), which suggests that only a handful of loci harbor meaningful *cis* asymmetry. Similar results have been reported in other autopolyploids (e.g., potato, sugarcane, alfalfa; Pham et al. 2017; Chen et al. 2020; Xue et al. 2022), consistent with dosage balance/stoichiometric constraints in autopolyploid expression. Preferential allele expression in such systems has been linked to high levels of genome heterogeneity (Pham et al. 2017) and may reflect subtle, locus-specific *cis* differences or local chromatin features rather than broad subgenome effects. Indeed, there is evidence that this phenomenon could be associated with allele-specific DNA methylation, variance in TE insertions, local chromatin structure and/or *cis*-regulatory element variation (Springer and Stupar 2007; Xue et al. 2022). Taken together, our results suggest that sainfoin possesses an autotetraploid origin that carries modest haplotypic divergence, without the system-wide expression dominance typical of neo-allopolyploids. However, allotetraploidy with closely related progenitor species cannot be entirely excluded at this point.

In conclusion, we generated a phased, chromosome-scale reference nuclear genome, along with comprehensive gene models and methylation maps, for a genotype of the representative AAC Mountainview cultivar. This assembly provides improved haplotype resolution and repeat continuity, as well as expanded gene annotation, compared to the previously sequenced genome of an undisclosed sainfoin cultivar/accession (He et al. 2024). In addition, we similarly produced a full-length chloroplast genome sequence for the AAC Mountainview genotype, as well as the first mitochondrial genome sequence for this species. We also delivered a tissue-enriched atlas that highlights pathways such as CT biosynthesis in young leaves, and elucidated both conserved and stress-specific transcriptomic changes under drought and waterlogging stress. Furthermore, we found that sainfoin exhibits balanced transcriptional regulation among alleles, suggesting an autotetraploid nature. These data provide a strong framework for downstream sainfoin breeding efforts, which will facilitate the agronomic improvement of this promising forage crop.

## Supplementary Information

Below is the link to the electronic supplementary material.

Supplementary file1 (XLSX 1982 KB)

Supplementary file2 (XLSX 1231 KB)

Supplementary file3 (XLSX 3151 KB)

Supplementary file4 (XLSX 64 KB)

Supplementary file5 (XLSX 561 KB)

Supplementary file6 (PDF 7437 KB)

Supplementary file7 (PDF 215 KB)

## Data Availability

All raw sequencing data have been deposited to the NCBI SRA under BioProject accession PRJNA1309166. Genome assemblies, annotations, and supplementary materials are also available in the Phytozome database (https://phytozome-next.jgi.doe.gov/info/962).

## Abbreviations

CTs: Condensed tannins
DEGs: Differentially expressed genes
LAI: LTR assembly index
LTR: Long terminal repeat
TEs: Transposable elements
