# Supplementary material for: A chromosome-scale reference genome and integrative transcriptome provide insight into tissue- and stress-specific responses in tetraploid sainfoin (Onobrychis viciifolia)
Source: Planta. 2026 May 14;263(6):155. doi: 10.1007/s00425-026-05021-y (PMC13176080; doi:10.1007/s00425-026-05021-y)
Supplement: Supplementary file 6 — Supplementary file6 (PDF 7437 KB) [file 425_2026_5021_MOESM6_ESM.pdf]

Supplementary Figures

A chromosome-scale reference genome and integrative transcriptome provide insight into tissue- and stress-specific responses in tetraploid sainfoin *(Onobrychis viciifolia)*

Cuong V. Nguyen, Dustin Cram, Halim Song, Rodrigo Ortega Polo, Hari Poudel, Bill Biligetu, Kimberley Burton Hughes, Surya Acharya, David Konkin*, Stacy D. Singer*

Corresponding authors

* Stacy D. Singer

Agriculture and Agri-Food Canada, Lethbridge Research and Development Centre, Lethbridge, AB, T1J 4B1, Canada

Email: stacy.singer@agr.gc.ca

ORCID: https://orcid.org/0000-0002-6973-3881

* David Konkin

National Research Council of Canada, Aquatic Crop Resource Development, Saskatoon, SK, S7N 0W9, Canada

Email: david.konkin@nrc-cnrc.gc.ca

ORCID: https://orcid.org/0000-0001-5410-8357

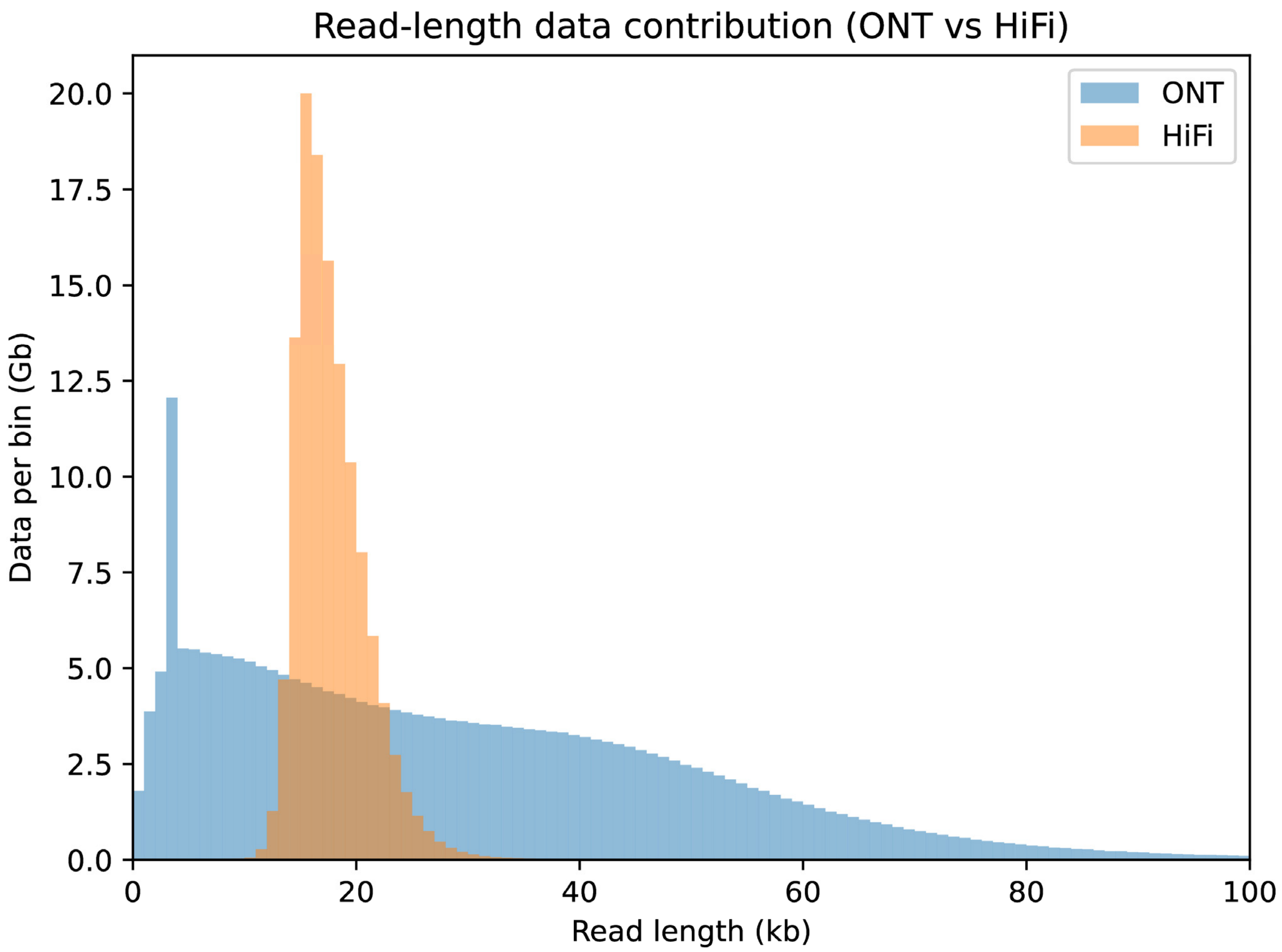

Fig. S1 Read-length data contribution for Oxford Nanopore (ONT) and PacBio HiFi sequencing reads for the sainfoin Mountainview genotype. Histograms were generated from raw FASTQ files, binned in 1 kb intervals, and plotted up to 100 kb read length, with the y-axis showing the total bases (Gb) contributed by reads in each bin (approximated by bin midpoint x read count)

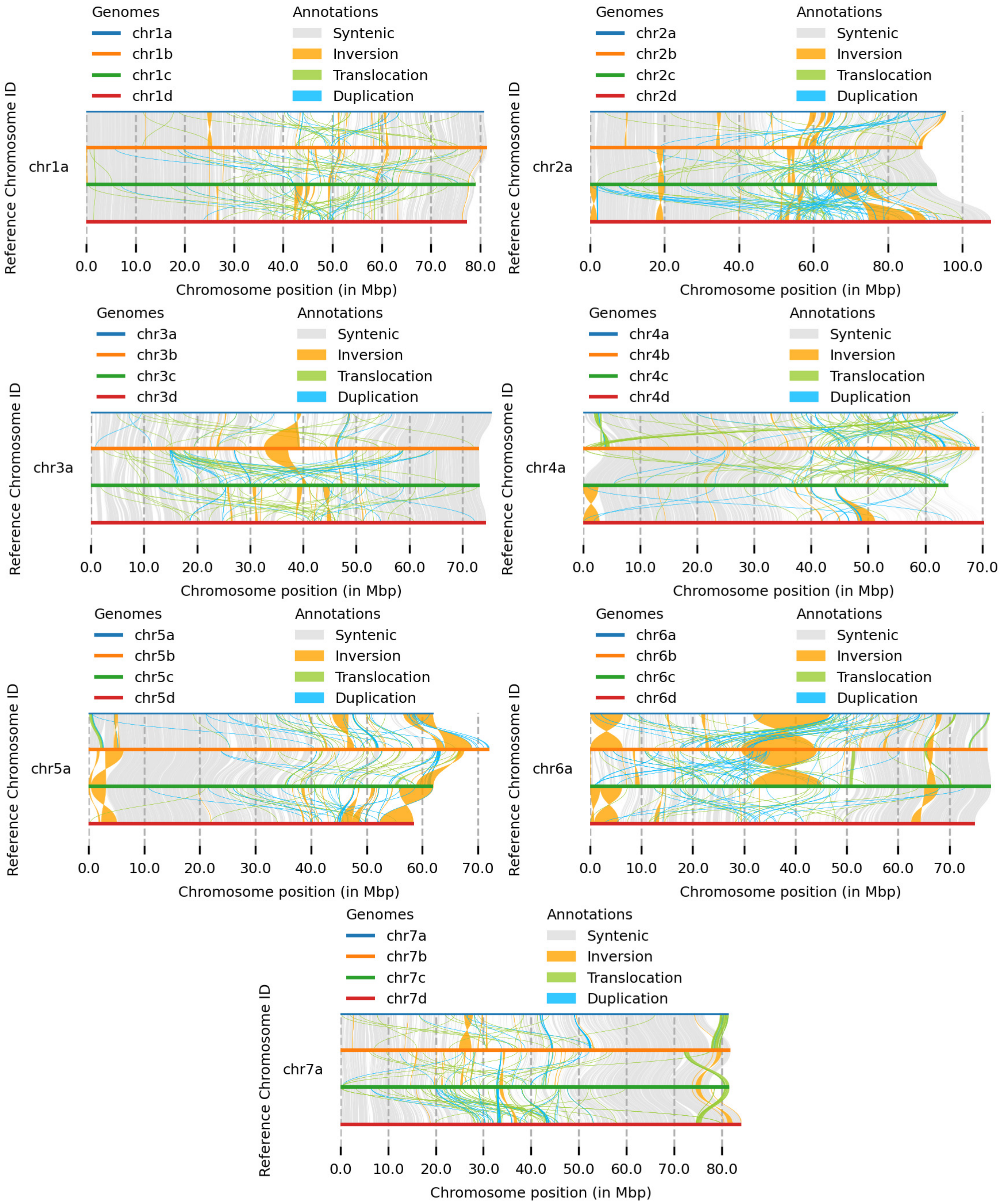

Fig. S2 Synteny and structural variation between homologous chromosomes of AAC Mountainview sainfoin. Colored lines represent syntenic blocks (gray) and structural rearrangements, including inversions (yellow), translocations (green), and duplications (blue)

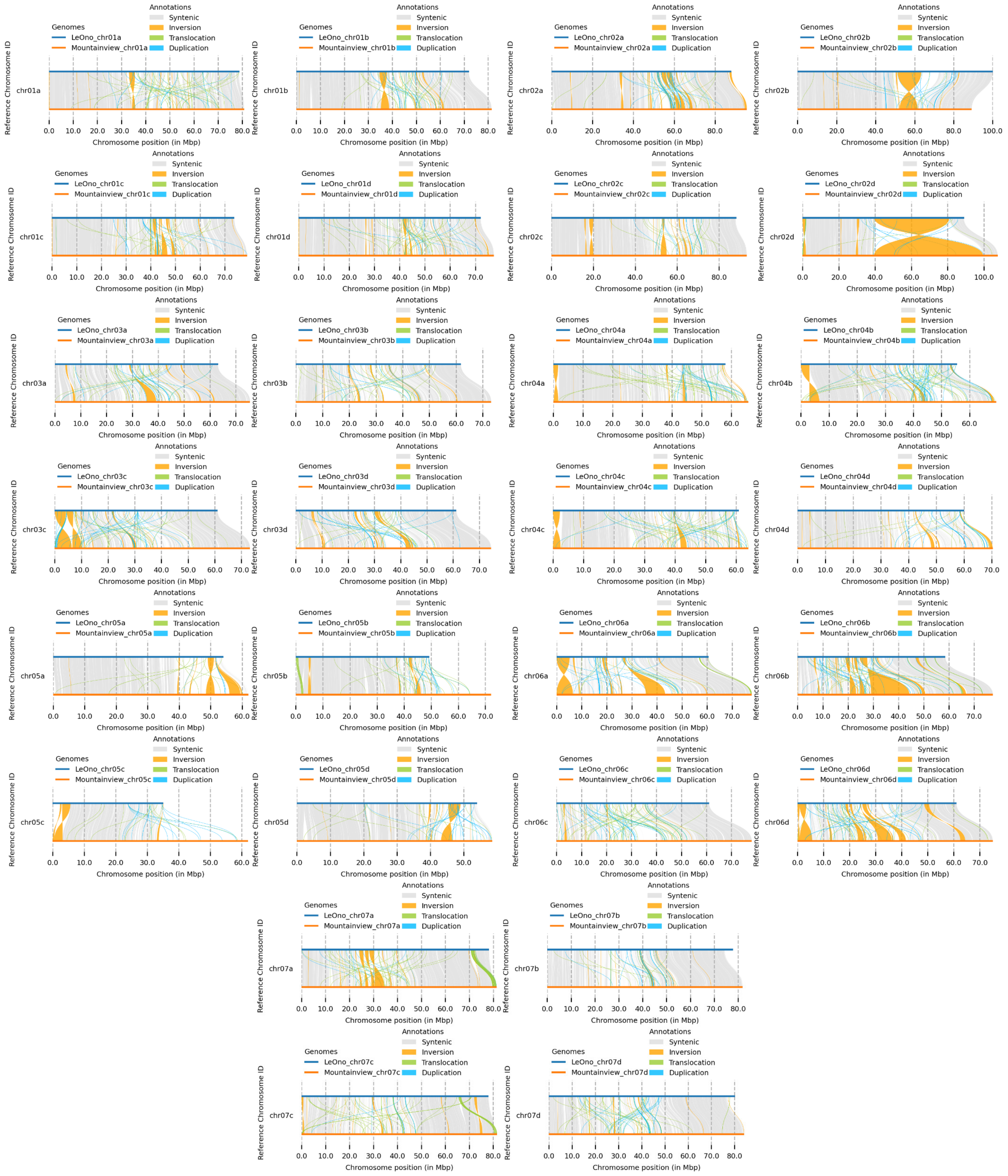

Fig. S3 Synteny and structural variation between sainfoin AAC Mountainview and He et al. (2024) genomes. Each panel shows pairwise alignments between homologous chromosome sets (chr1–chr7), with AAC Mountainview haplotypes plotted below the He et al. haplotypes. Gray lines indicate syntenic regions, while structural rearrangements are highlighted as inversions (orange), translocations (green), and duplications (blue)

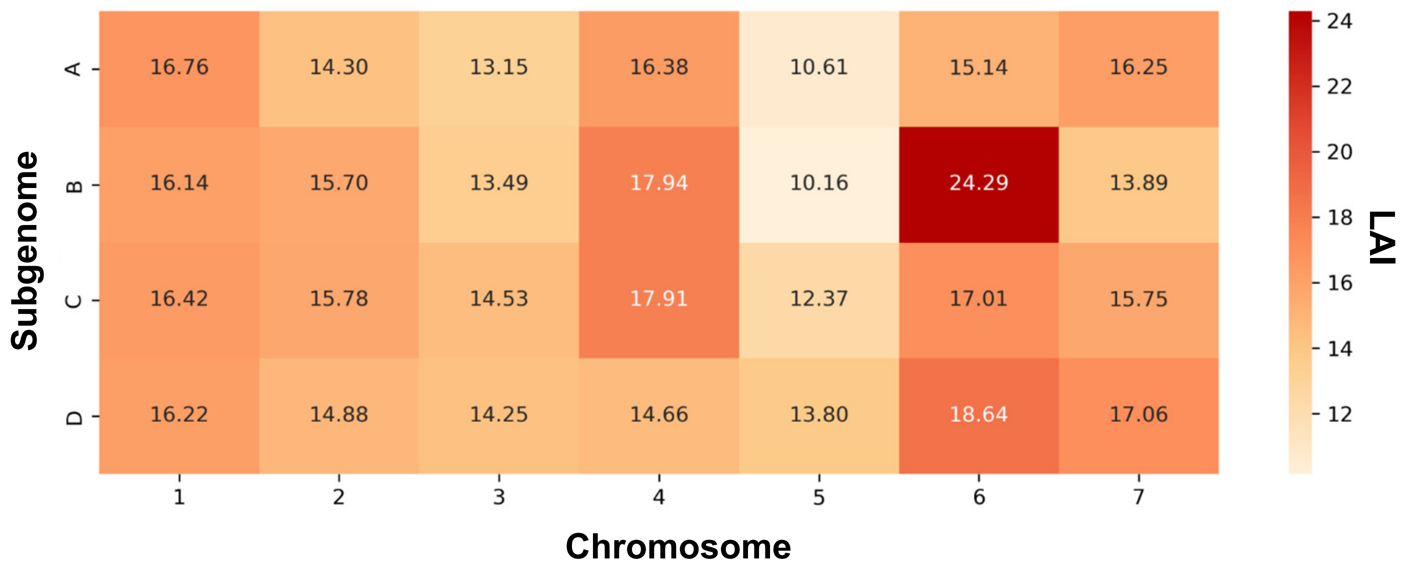

Fig. S4 LTR Assembly Index (LAI) heatmap across chromosomes. LAI values are shown for each of the four haplotypes (A–D) of the seven base chromosomes. Higher values (darker shading) indicate greater assembly continuity of LTR retrotransposons

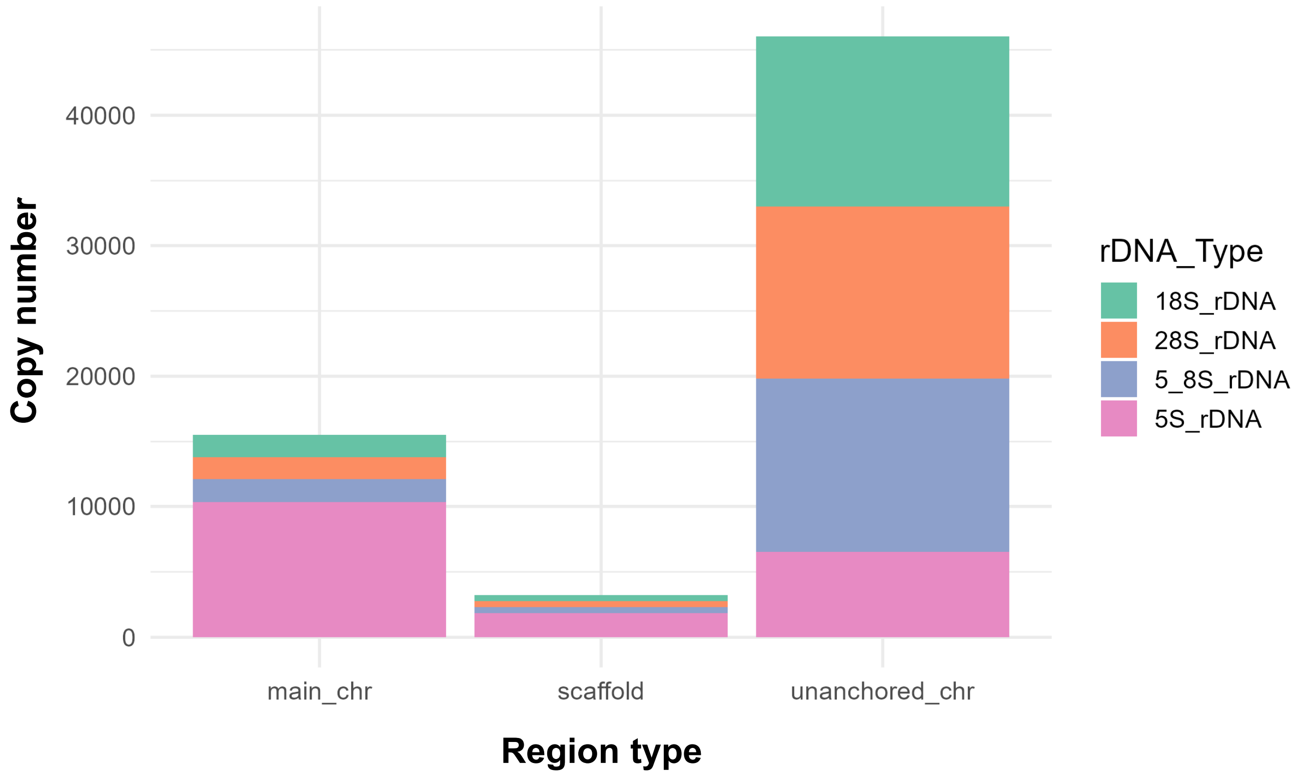

Fig. S5 Genomic distribution and clustering of rDNA arrays. Copy numbers of the four major rDNA types (18S, 28S, 5.8S, and 5S) are partitioned by genomic region: chromosome-anchored sequences (main_chr), minor scaffolds, and chromosome-assigned unanchored scaffolds

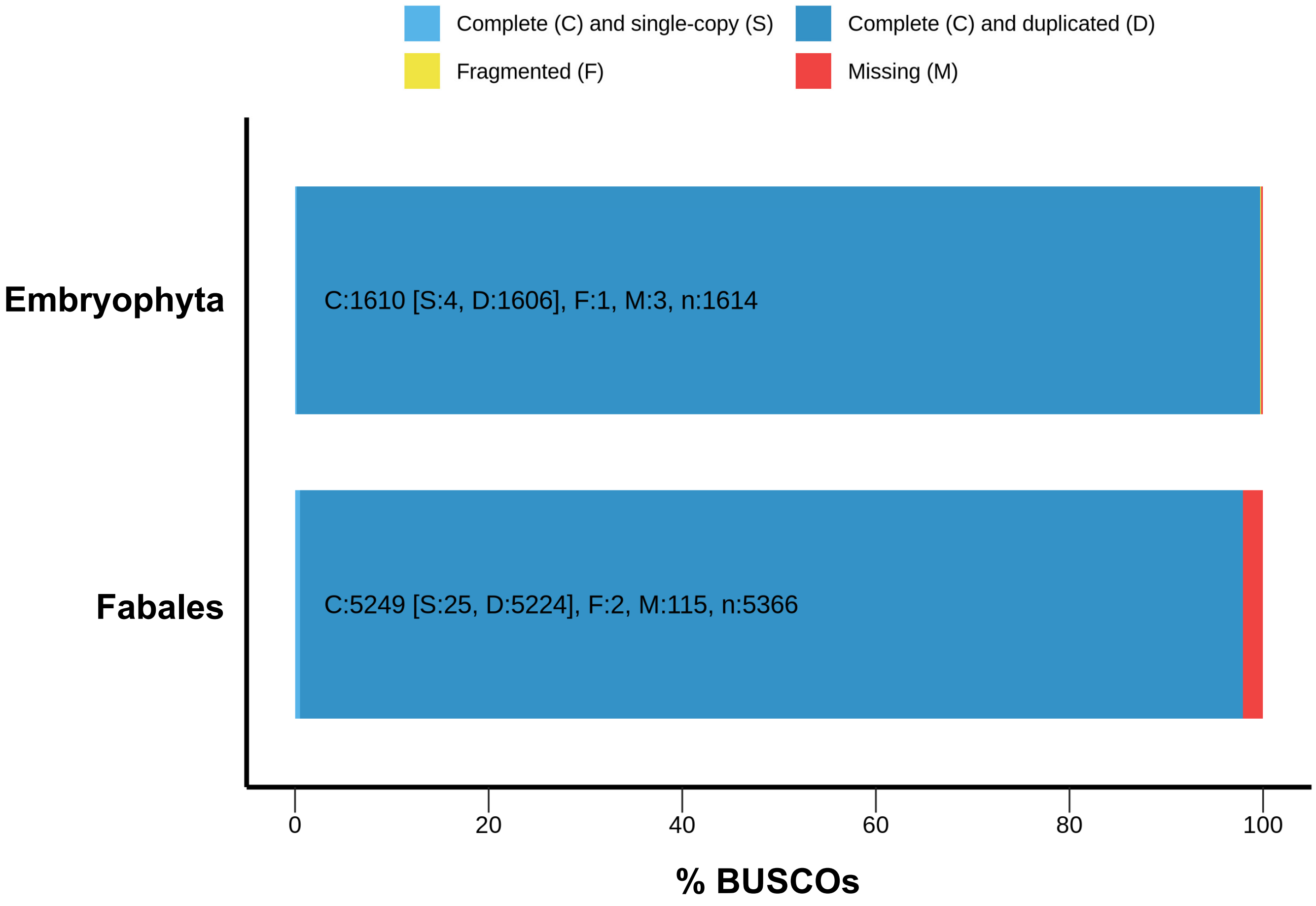

Fig. S6 BUSCO assessment of gene annotation completeness using the Fabales and Embryophyta ortholog datasets. C, Complete BUSCOs; S, complete and single-copy BUSCOs; D, complete and duplicated BUSCOs; F, fragmented BUSCOs; M, missing BUSCOs

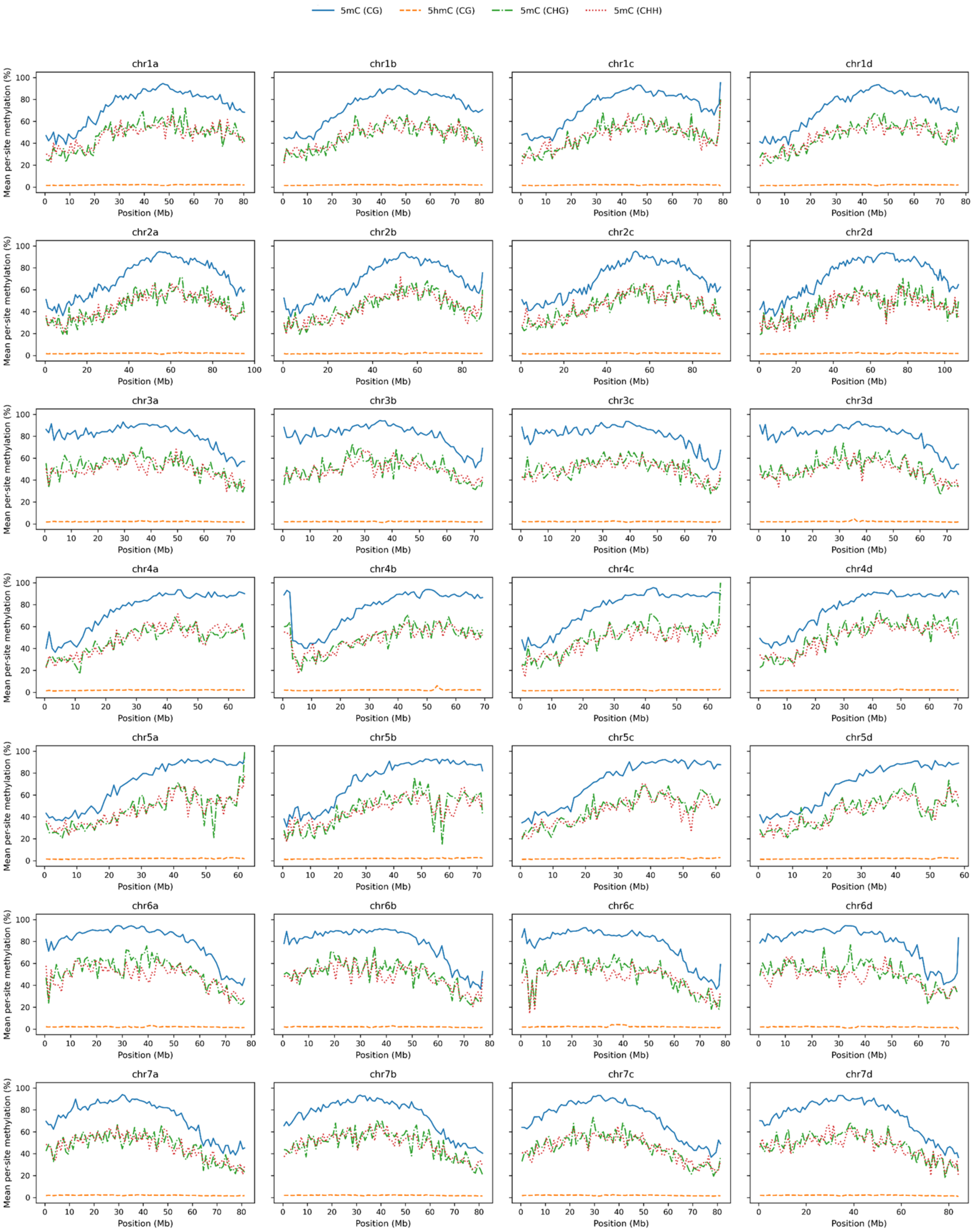

Fig. S7 Genome-wide distribution of 5-methylcytosine (mCG, mCHG, mCHH) and 5-hydroxymethylcytosine (5hmCG) levels across 1 Mb windows in sainfoin. Mean per-site methylation fractions (%) are shown for non-overlapping 1 Mb windows across each chromosome

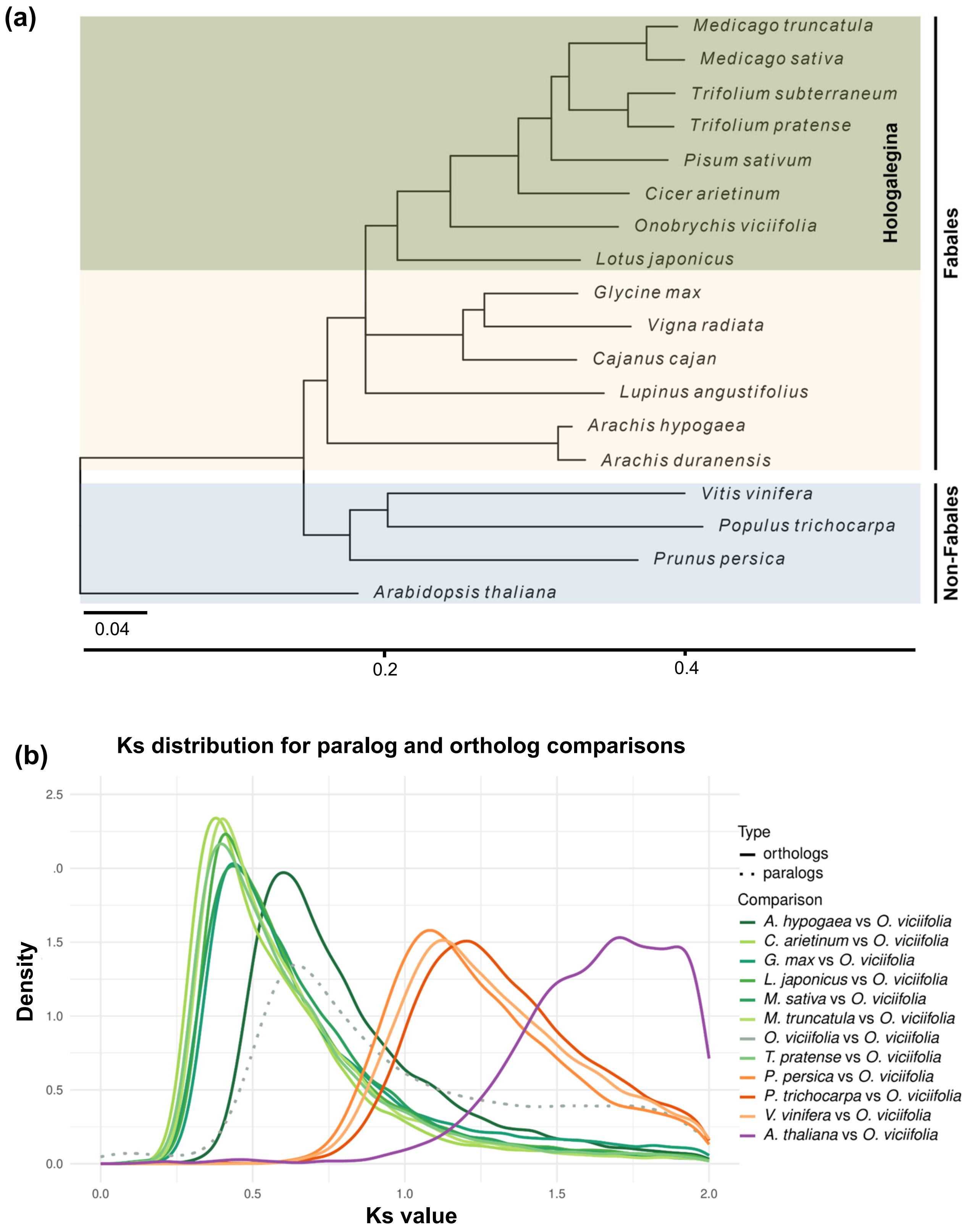

Fig. S8 Phylogenetic placement and whole-genome duplication history of *O. viciifolia*. (a) Maximum likelihood species tree inferred from high-occupancy orthogroups using OrthoFinder. *O. viciifolia* is positioned within the Hologalegina, consistent with legume phylogeny. (b) K_s distribution of synonymous substitutions among syntenic gene pairs. Solid lines represent ortholog comparisons between *O. viciifolia* and other legume and outgroup species; the dashed line indicates within-species *O. viciifolia* paralogs. Peaks near $K_s \approx 0.4$-0.6 correspond to a legume-wide whole-genome duplication. Peaks at $K_s > 1.25$ present in outgroup comparisons represents the ancestral ploidy event

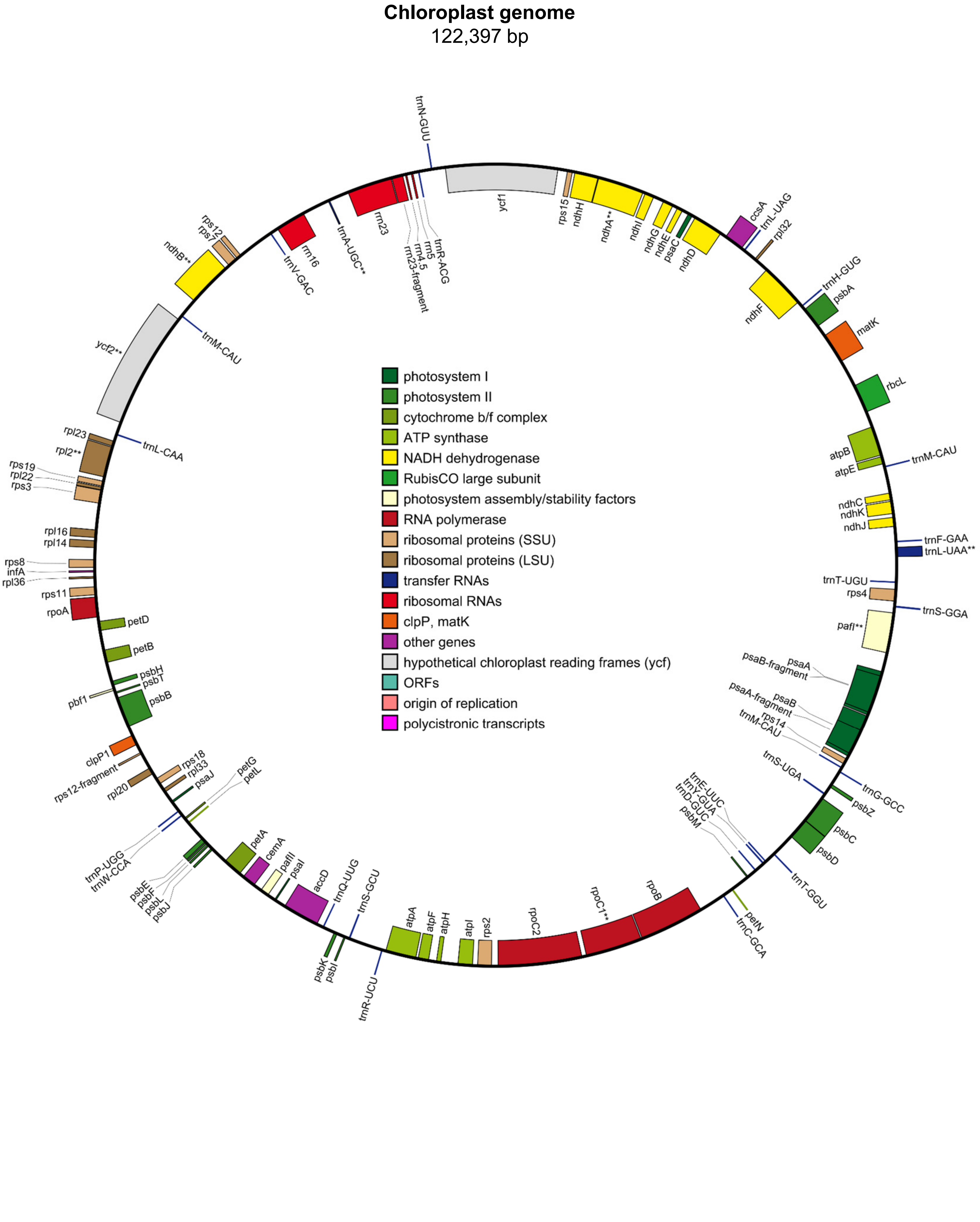

Fig. S9 Annotation of the AAC Mountainview chloroplast genome. clpP, proteolytic subunit of CLP protease; LSU, large subunit; matK, maturase K; ORF, open reading frame; SSU, small subunit

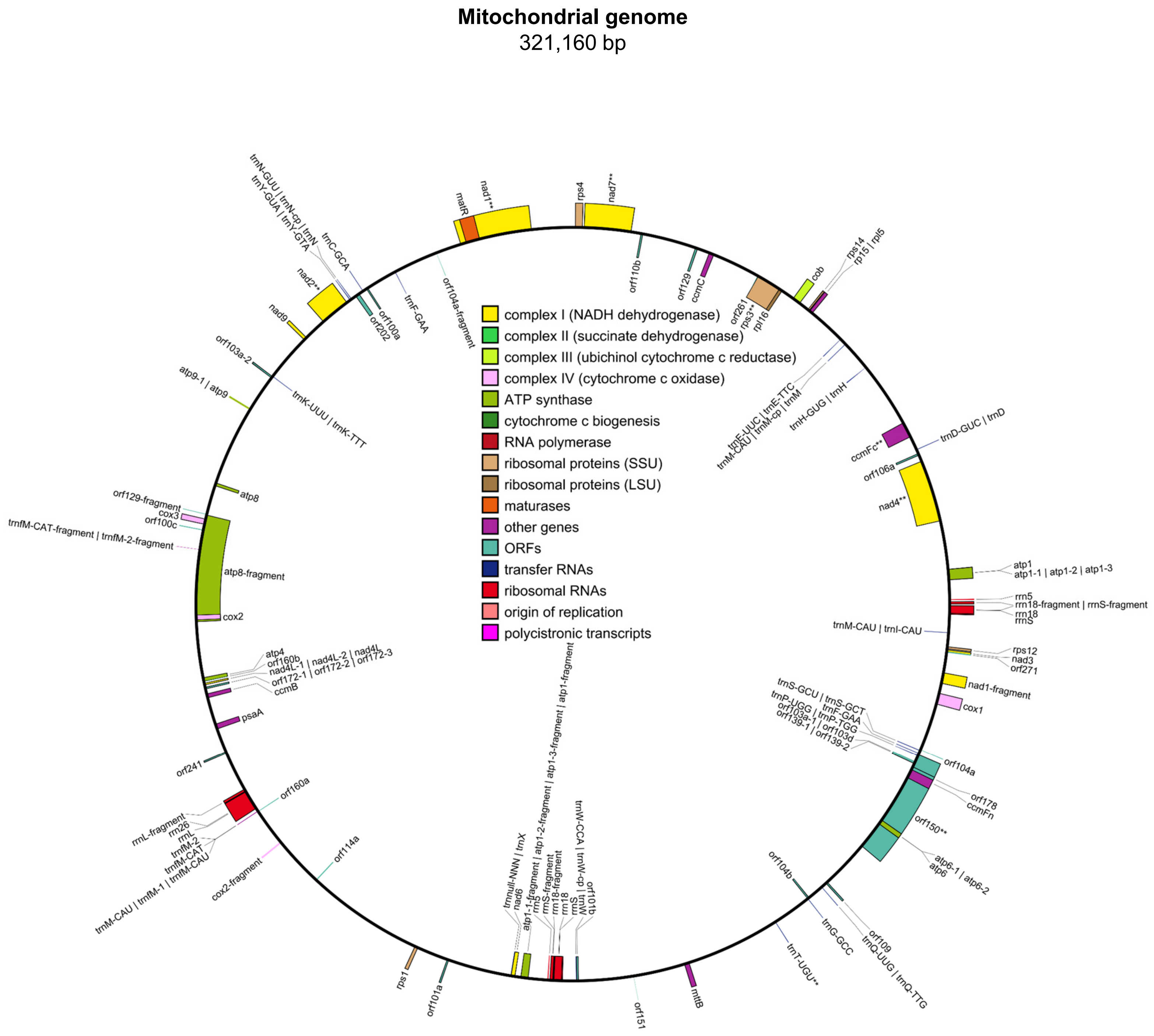

Fig. S10 Annotation of the AAC Mountainview mitochondrial genome. LSU, large subunit; ORF, open reading frame; SSU, small subunit

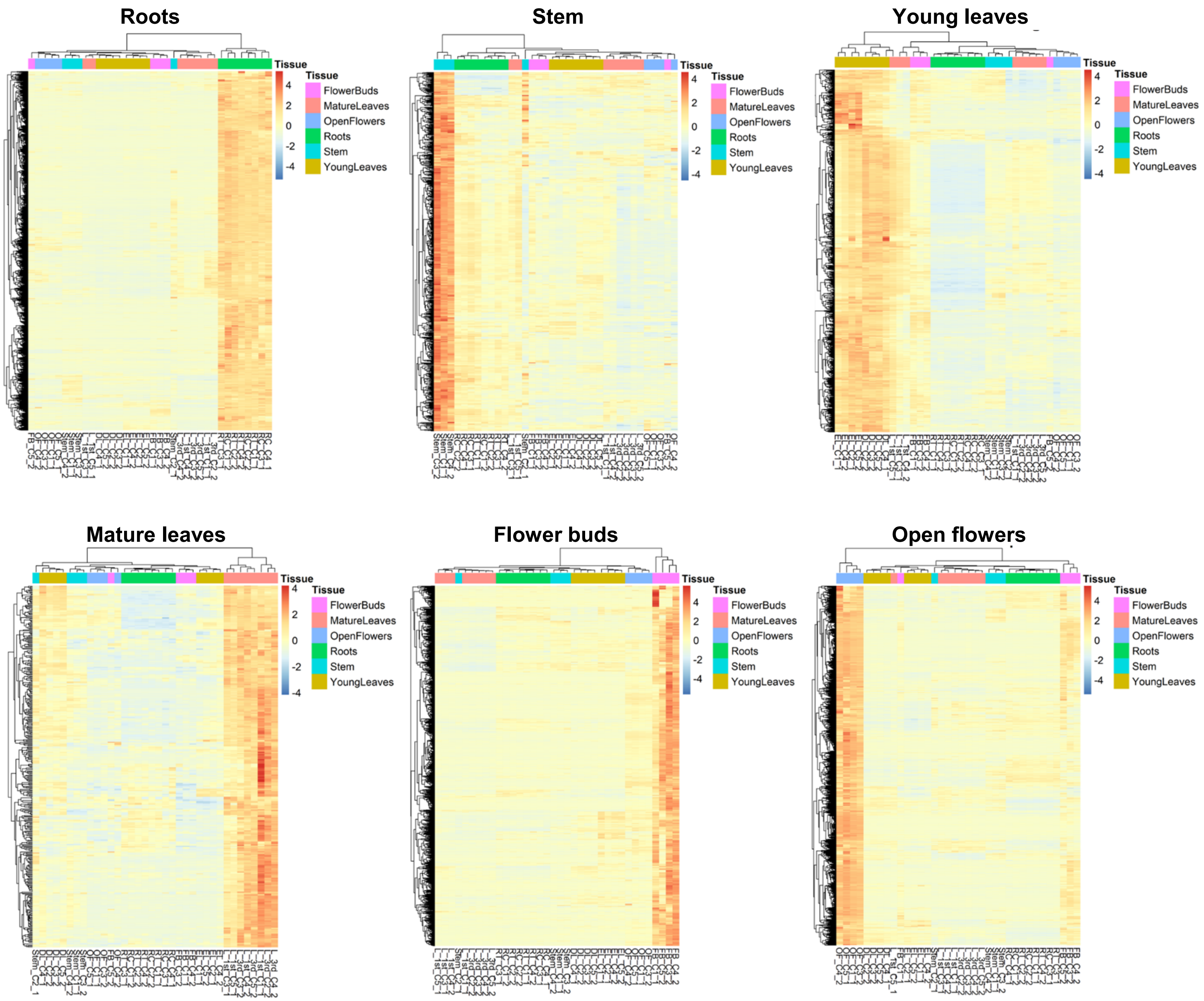

Fig. S11 Tissue-enriched gene expression heatmaps across six sainfoin tissue types. Each heatmap shows the expression of genes enriched in one tissue types across all six tissue types (roots, stem, young leaves, mature leaves, flower buds, open flowers). For enrichment analysis, closely related tissues were collapsed into broader categories (developing leaves + emerging leaves = young leaves, first fully expanded leaves + third fully expanded leaves = mature leaves, root crown + root tips = roots). Heatmaps display variance-stabilized expression levels (VST) with row scaling (Z-scores)

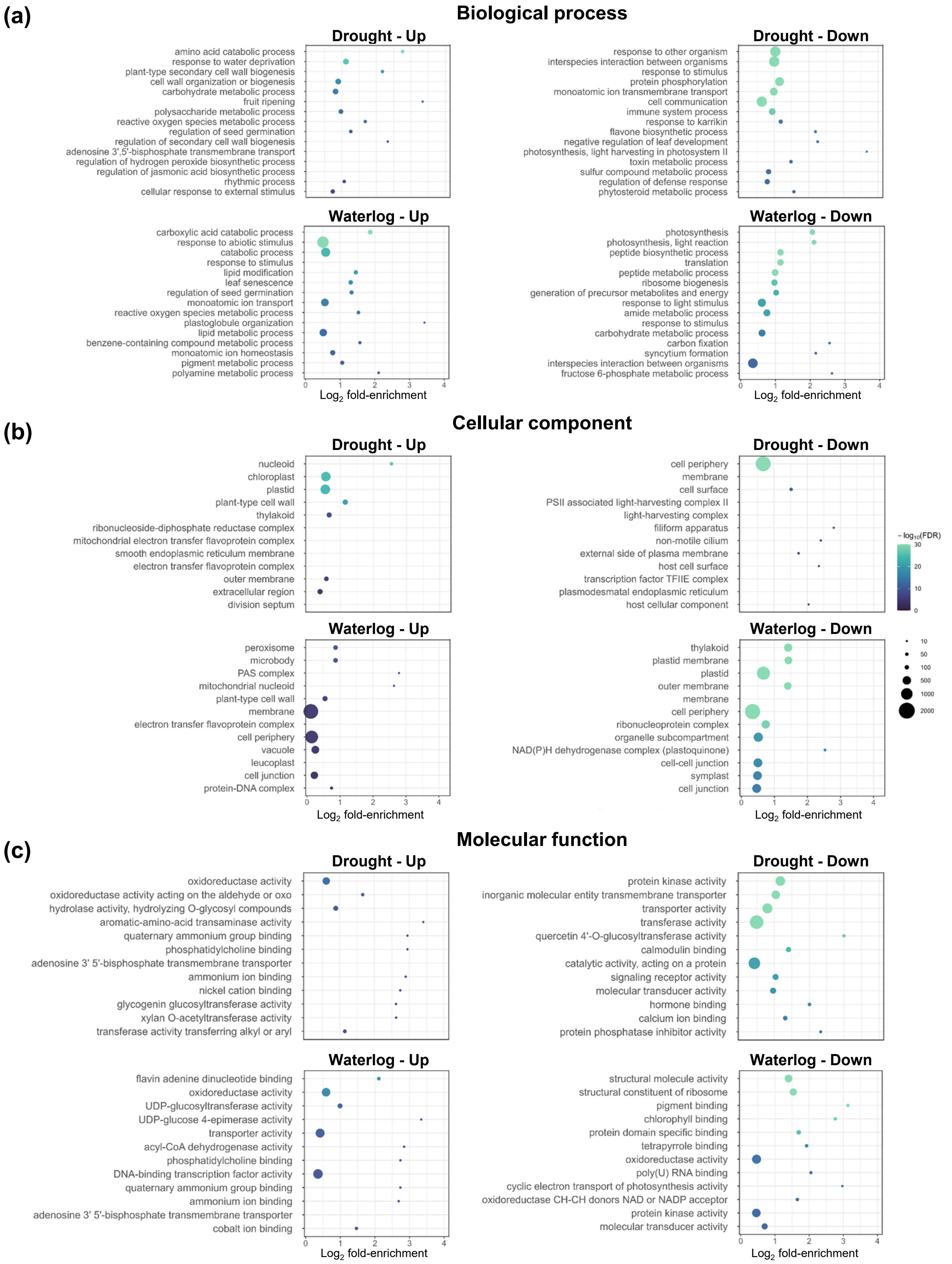

Fig. S12 GO term analysis enrichment of transcriptomic changes incurred under drought and waterlogging compared to control conditions, respectively, in AAC Mountainview sainfoin leaves. (a) Biological process category, (b) cellular component category, and (c) molecular function category. Analyses were conducted using a significance level of 0.05

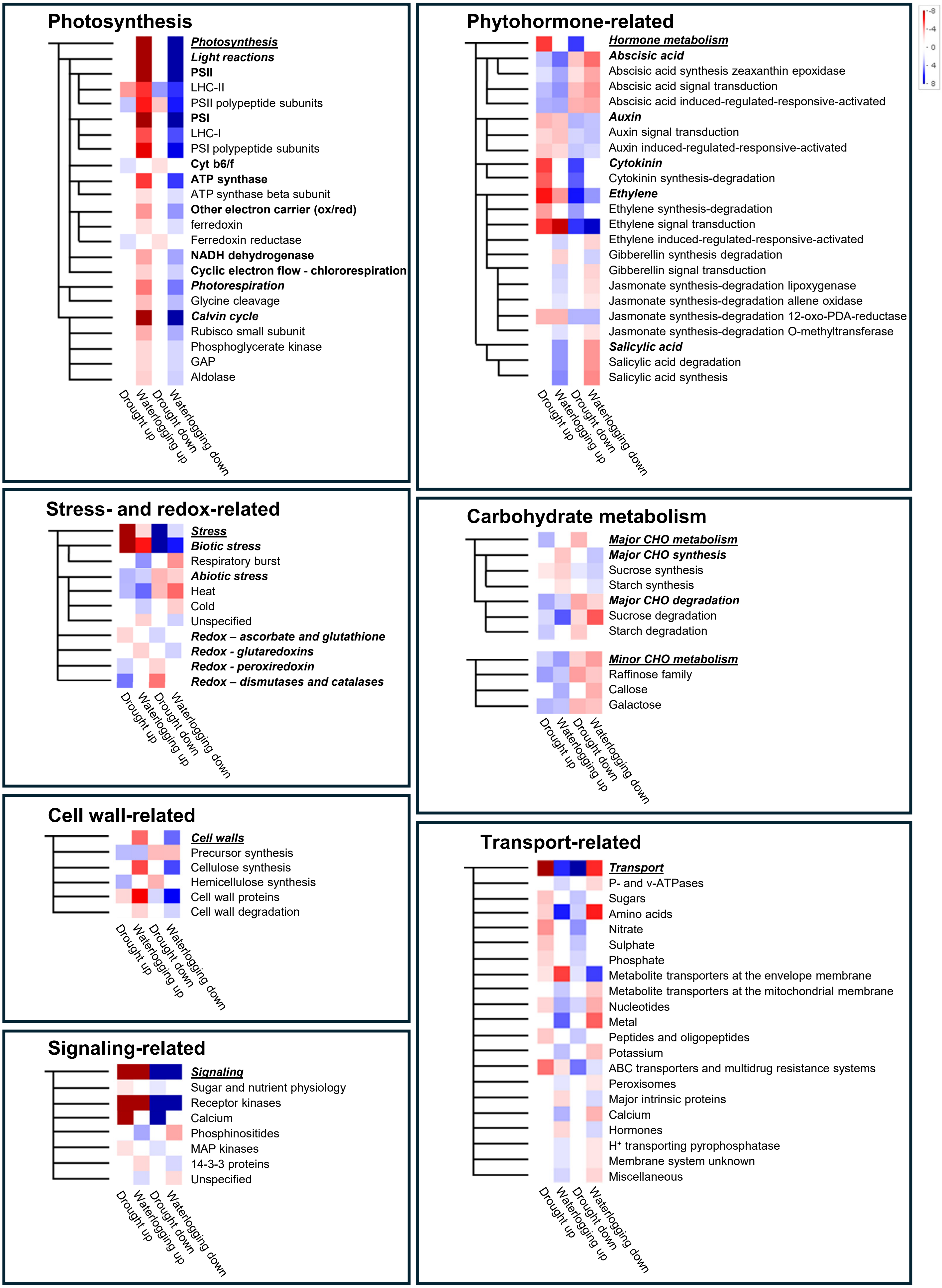

Fig. S13 PageMan enrichment analysis of DEGs under drought and waterlogged conditions for stress-related pathways. Bold and italic terms indicate higher level nodes in pathways. CHO, carbohydrate; PS, photosystem; LHC, light-harvesting complex II; cytb6/f, cytochrome b6/f

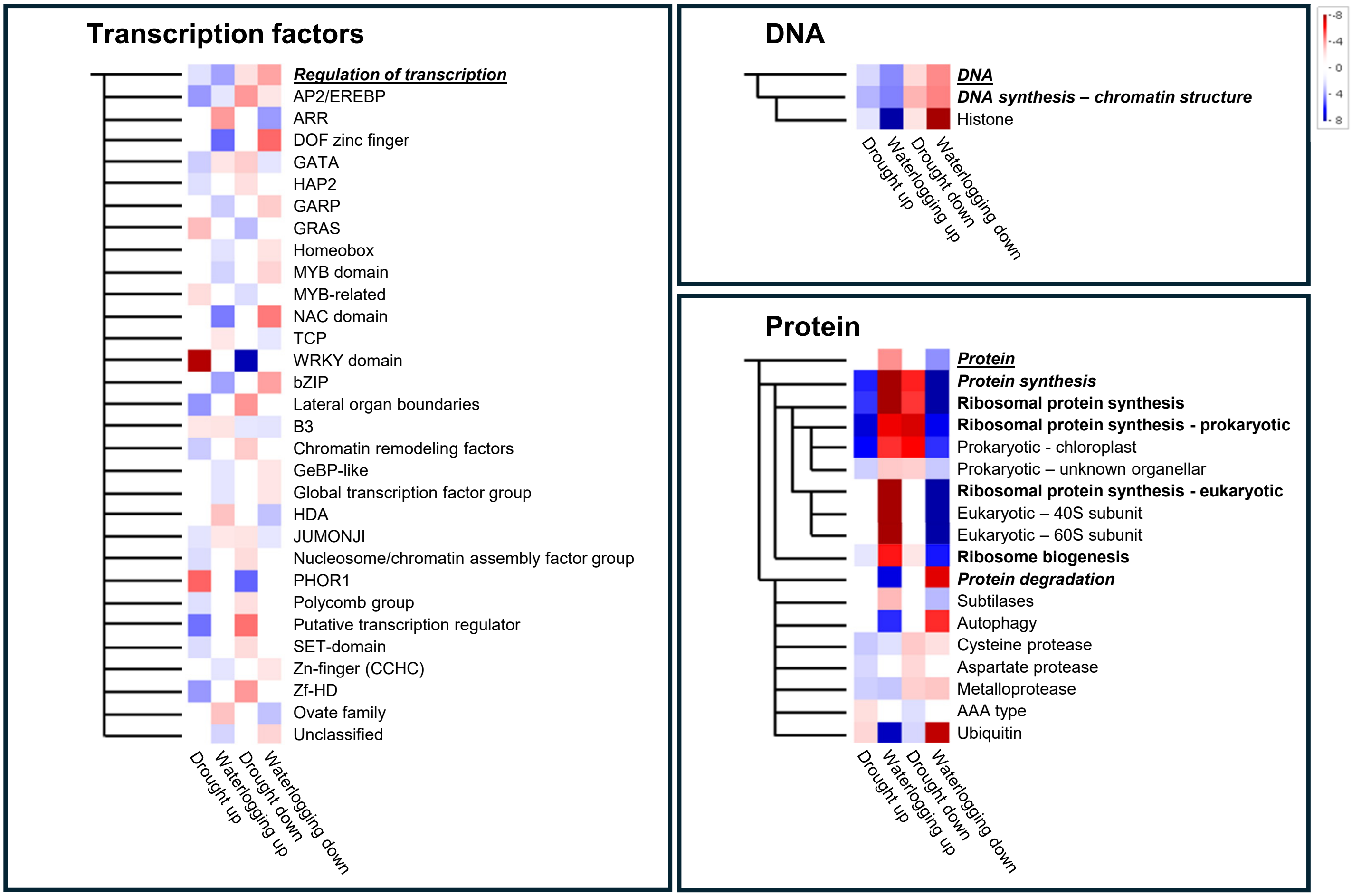

Fig. S14 PageMan enrichment analysis of DEGs under drought and waterlogged conditions for transcription factor-, DNA-, and protein-related terms. Bold and italic terms indicate higher level nodes in pathways
