# Supplementary material for: A chromosome-scale reference genome and integrative transcriptome provide insight into tissue- and stress-specific responses in tetraploid sainfoin (Onobrychis viciifolia)
Source: Planta. 2026 May 14;263(6):155. doi: 10.1007/s00425-026-05021-y (PMC13176080; doi:10.1007/s00425-026-05021-y)
Supplement: Supplementary file 7 — Supplementary file7 (PDF 215 KB) [file 425_2026_5021_MOESM7_ESM.pdf]

Supplementary Tables

A chromosome-scale reference genome and integrative transcriptome provide insight into tissue- and stress-specific responses in tetraploid sainfoin *(Onobrychis viciifolia)*

Cuong V. Nguyen, Dustin Cram, Halim Song, Rodrigo Ortega Polo, Hari Poudel, Bill Biligetu, Kimberley Burton Hughes, Surya Acharya, David Konkin*, Stacy D. Singer*

Corresponding authors

* Stacy D. Singer

Agriculture and Agri-Food Canada, Lethbridge Research and Development Centre, Lethbridge, AB, T1J 4B1, Canada

Email: stacy.singer@agr.gc.ca

ORCID: https://orcid.org/0000-0002-6973-3881

* David Konkin

National Research Council of Canada, Aquatic Crop Resource Development, Saskatoon, SK, S7N 0W9, Canada

Email: david.konkin@nrc-cnrc.gc.ca

ORCID: https://orcid.org/0000-0001-5410-8357

Table S1 Genes in the flavonoid biosynthetic pathway used for homology and expression analyses

Gene	Reference species	Gene ID	Encoded protein
PAL	*A. thaliana*	AT2G37040	Phenylalanine ammonia-lyase
C4H	*A. thaliana*	AT2G30490	Cinnamate 4-hydroxylase
4CL	*A. thaliana*	AT3G21230	4-coumarate CoA ligase
CHS	*A. thaliana*	AT5G13930	Chalcone synthase
CHI	*A. thaliana*	AT3G55120	Chalcone isomerase
F3H	*A. thaliana*	AT3G51240	Flavanone 3-hydroxylase
F3'5'H	*V. vinifera*	NP_001268157.1	Flavonoid 3',5'-hydroxylase
DFR	*A. thaliana*	AT5G42800	Dihydroflavonol reductase
ANS/LDOX	*A. thaliana*	AT4G22880	Anthocyanidin synthase
ANR	*V. vinifera*	NP_001267885.1	Anthocyanidin reductase
LAR	*V. vinifera*	NP_001267887.1	Leucoanthocyanidin reductase
UGT	*A. thaliana*	AT4G15280	UDP-glycosyltransferase
OMT	*A. thaliana*	AT5G54160	O-methyltransferase
AHA10	*A. thaliana*	AT1G17260	Plasma membrane H^+-ATPase 10
LAC15	*A. thaliana*	AT5G48100	Laccase-like 15
TT2	*A. thaliana*	AT5G35550	MYB transcription factor
TT8	*A. thaliana*	AT4G09820	bHLH transcription factor
TTG1	*A. thaliana*	AT5G24520	WD40 transcription factor
MATE	*A. thaliana*	AT3G59030	Flavonoid transporter (TT12-like)
MYB12	*A. thaliana*	AT2G47460	MYB transcription factor

A. thaliana, *Arabidopsis thaliana*; *V. vinifera*, *Vitis vinifera*

Table S2 Sequencing data summary for the AAC Mountainview sainfoin reference genome

Platform	Source	Library	Reads / Pairs	Yield (Gb)	Mean length (bp)	N50 (bp)	Coverage (×)*
Illumina WGS	Whole cell	PE150	700,009,338 pairs	210	150	150	365×
PacBio HiFi	Nuclei	HiFi CCS	7,124,735 reads	123.4	17,315	17,187	214×
Oxford Nanopore	Whole cell	Long reads (simplex + duplex)	23,677,414 reads	245.6	10,371	25,302	426×
Hi-C	Whole cell	PE150	439,536,585 pairs	132.1	150-151	150-151	–

*Coverage computed against haploid genome size = 575.8 Mb

Table S3 Summary statistics of haplotype-resolved assembly: contigs, scaffolds, and final assembly

Metric	Contigs	Scaffolds	Final Assembly
No. of contigs	3,210	—	—
No. of scaffolds	—	109	109
Total size (bp)	2,338,123,456	2,363,951,495	2,363,917,914
N50	41.2 Mb	78.15 Mb	78.15 Mb
Max scaffold length (main chrs)	—	107.76 Mb	107.74 Mb
GC content (%)	36.17%	36.16%	36.18%
Gaps (Ns, %)	0.48%	0.49%	0.48%
BUSCO (Fabales, %)	97.70%	97.70%	97.70%
LAI	—	—	14.7

Table S4 BUSCO completeness assessment of the sainfoin genome and predicted gene models using the Fabales and Embryophyta lineage datasets

Input	Mode	Lineage	C (%)	S (%)	D (%)	F (%)	M (%)	BUSCOs
Whole Genome	genome	Emb	99.6	1.2	98.4	0.3	0.1	1,614
Whole Genome	genome	Fab	97.7	0.4	97.3	0.2	2.1	5,366
Subgenome A Genes	protein	Emb	97.1	70.3	26.8	0.4	2.5	1,614
Subgenome B Genes	protein	Emb	95.8	69.7	26.1	0.6	3.6	1,614
Subgenome C Genes	protein	Emb	95.0	70.2	24.8	0.7	4.3	1,614
Subgenome D Genes	protein	Emb	95.2	70.0	25.2	0.4	4.4	1,614
Whole Genome Genes	protein	Emb	99.7	0.2	99.5	0.1	0.2	1,614
Subgenome A Genes	protein	Fab	94.7	69.8	24.9	0.2	5.1	5,366
Subgenome B Genes	protein	Fab	93.0	68.8	24.2	0.2	6.8	5,366
Subgenome C Genes	protein	Fab	92.6	68.7	23.9	0.3	7.1	5,366
Subgenome D Genes	protein	Fab	92.5	68.8	23.7	0.1	7.4	5,366
Whole Genome Genes	protein	Fab	97.7	0.4	97.3	0	2.3	5,366

C, Complete BUSCOs; S, complete and single-copy BUSCOs; D, complete and duplicated BUSCOs; F, fragmented BUSCOs; M, missing BUSCOs

Table S5 Comparison of homolog numbers in flavonoid pathway gene families between sainfoin genomes

Gene family	AAC Mountainview genome	He et al. genome*
PAL	16	16
C4H	13	11
4CL	14	15
CHS	34	32
CHI	20	12
F3H	9	9
F3'5'H	12	12
DFR	10	12
ANS	4	3
ANR	8	7
LAR	4	4
UGT	19	18
OMT/COMT-like	29	22
AHA10-like	56	52
LAC/TT10-like	8	8
TT2	3	2
TT8	8	6
TTG1	10	12
MATE/TT12	17	15
MYB12	3	2

4CL, 4-coumarate:CoA ligase; AHA10-like, ATP-binding cassette transporter AHA10-like; ANR, anthocyanidin reductase; ANS, anthocyanidin synthase; C4H, cinnamate 4-hydroxylase; CHI, chalcone isomerase; CHS, chalcone synthase; DFR, dihydroflavonol reductase; F3H, flavanone 3-hydroxylase; F3′5′H, flavonoid 3′,5′-hydroxylase; LAC/TT10-like, laccase/TRANSPARENT TESTA10-like; LAR, leucoanthocyanidin reductase; MATE/TT12, multidrug and toxic compound extrusion transporter/TRANSPARENT TESTA12; MYB12, R2R3-MYB transcription factor MYB12; OMT/COMT-like, O-methyltransferase/caffeic acid O-methyltransferase-like; PAL, phenylalanine ammonia-lyase; TT2, R2R3-MYB transcription factor TRANSPARENT TESTA2; TT8, basic helix–loop–helix transcription factor TRANSPARENT TESTA8; TTG1, WD40 repeat protein TRANSPARENT TESTA GLABRA1; UGT, UDP-glycosyltransferase

* He et al. (2024) A chromosome-level genome assembly for *Onobrychis viciifolia* reveals gene copy number gain underlying enhanced proanthocyanidin biosynthesis. Commun Biol 7:19

Table S6 LTR Assembly Index (LAI) scores for each chromosome and genome

Chr (full)	Chromosome	Haplotype	LAI	n
chr1a	chr1	A	16.76	261
chr1b	chr1	B	16.14	263
chr1c	chr1	C	16.42	255
chr1d	chr1	D	16.22	249
chr2a	chr2	A	14.30	310
chr2b	chr2	B	15.70	289
chr2c	chr2	C	15.78	302
chr2d	chr2	D	14.88	351
chr3a	chr3	A	13.15	243
chr3b	chr3	B	13.49	235
chr3c	chr3	C	14.53	236
chr3d	chr3	D	14.25	240
chr4a	chr4	A	16.38	211
chr4b	chr4	B	17.94	223
chr4c	chr4	C	17.91	205
chr4d	chr4	D	14.66	226
chr5a	chr5	A	10.61	198
chr5b	chr5	B	10.16	232
chr5c	chr5	C	12.37	198
chr5d	chr5	D	13.8	187
chr6a	chr6	A	15.14	251
chr6b	chr6	B	24.29	250
chr6c	chr6	C	17.01	252
chr6d	chr6	D	18.64	242
chr7a	chr7	A	16.25	263
chr7b	chr7	B	13.89	264
chr7c	chr7	C	15.75	264
chr7d	chr7	D	17.06	272
**Whole genome**			**14.65**	

n, number of intact LTR retrotransposons

Table S7 Distribution and counts of rDNA types across anchored chromosomes and unanchored scaffolds

Chromosome	5_8S_rDNA	5S_rDNA	18S_rDNA	28S_rDNA
chr1a	1	0	0	0
chr1b	1	0	0	0
chr1c	1	4	0	0
chr1d	0	2	0	0
chr2c	1	0	1	0
chr2d	0	0	0	1
chr3a	0	2,330	0	0
chr3b	0	1,041	0	0
chr3c	0	1,600	0	0
chr3d	0	813	0	0
chr4a	18	1,620	10	12
chr4b	30	1,156	27	31
chr4c	9	627	7	7
chr4d	306	1,148	304	306
chr5a	274	0	270	275
chr5b	557	1	566	567
chr5c	269	0	265	260
chr5d	269	0	257	263
chr6a	1	2	1	1
chr6b	2	2	1	0
chr6c	1	2	0	0
chr6d	1	1	1	0
chr7a	1	0	0	0
chr3un	0	3,122	0	0
chr4un	0	3,410	0	0
chr5un	13,283	0	12,989	13,215
scaffold_101	22	0	22	22
scaffold_1019	6	0	6	6
scaffold_102	22	0	22	22
scaffold_120	20	0	20	19
scaffold_1248	5	0	6	5
scaffold_1307	5	0	5	6
scaffold_1380	4	0	4	4
scaffold_142	18	0	17	18
scaffold_1490	4	0	4	4
scaffold_170	14	0	14	13
scaffold_179	13	0	13	13
scaffold_1902	4	0	4	4
scaffold_2002	3	0	3	3

scaffold_2046	3	0	3	3
scaffold_207	13	0	13	13
scaffold_209	13	0	13	14
scaffold_2099	3	0	3	3
scaffold_213	13	0	13	12
scaffold_2319	0	90	0	0
scaffold_2336	0	88	0	0
scaffold_2423	0	71	0	0
scaffold_2461	0	83	0	0
scaffold_2509	2	0	2	2
scaffold_261	11	0	11	12
scaffold_2669	0	65	0	0
scaffold_2701	2	0	2	2
scaffold_2712	0	60	0	0
scaffold_2719	0	60	0	0
scaffold_2727	0	56	0	0
scaffold_2733	0	57	0	0
scaffold_2737	0	56	0	0
scaffold_2742	0	56	0	0
scaffold_2744	0	55	0	0
scaffold_2754	0	51	0	0
scaffold_2758	0	51	0	0
scaffold_2762	0	49	0	0
scaffold_2768	0	50	0	0
scaffold_2770	0	49	0	0
scaffold_2771	0	49	0	0
scaffold_2772	0	49	0	0
scaffold_2776	0	49	0	0
scaffold_2780	0	47	0	0
scaffold_2785	0	45	0	0
scaffold_2791	0	45	0	0
scaffold_2792	0	44	0	0
scaffold_2800	0	43	0	0
scaffold_2801	0	43	0	0
scaffold_2804	0	43	0	0
scaffold_2805	0	42	0	0
scaffold_2809	0	40	0	0
scaffold_2813	0	40	0	0
scaffold_2821	0	39	0	0
scaffold_2823	0	38	0	0
scaffold_2836	0	30	0	0
scaffold_2838	0	29	0	0
scaffold_2840	0	27	0	0

scaffold_2841	0	27	0	0
scaffold_2846	0	18	0	0
scaffold_303	11	0	11	11
scaffold_342	10	0	10	10
scaffold_37	61	0	60	60
scaffold_373	9	0	9	9
scaffold_379	9	0	9	9
scaffold_44	45	0	45	45
scaffold_49	39	0	39	38
scaffold_702	6	0	6	6
scaffold_74	28	0	28	28
scaffold_77	27	0	27	27
scaffold_88	23	0	23	24

Table S8 Gene model statistics (number of genes, exons, introns, coding sequence length, isoforms) per chromosome

Chrom	Total Length (bp)	Genes	Transcripts	Avg Isoforms Per Gene	Exons	Avg Exons per Transcript	Avg CDS Length Per Transcript (bp)	Avg Exon Length (bp)	Introns	Avg Intron Length (bp)
chr1a	80,795,861	4,511	5,597	1.24	30,629	5.47	1,325.1	242.1	25,032	493.6
chr1b	81,467,897	4,463	5,447	1.22	30,102	5.53	1,347.1	243.8	24,655	508.3
chr1c	79,052,274	4,483	5,484	1.22	29,934	5.46	1,332.9	244.2	24,450	495.6
chr1d	77,355,037	4,505	5,502	1.22	29,725	5.40	1,306.3	241.8	24,223	501.0
chr2a	95,601,905	6,077	7,378	1.21	39,653	5.37	1,321.8	245.9	32,275	473.1
chr2b	89,219,306	6,136	7,419	1.21	39,882	5.38	1,333.7	248.1	32,463	467.7
chr2c	93,192,014	6,264	7,575	1.21	39,742	5.25	1,305.7	248.9	32,167	470.4
chr2d	107,743,414	6,223	7,503	1.21	39,486	5.26	1,310.0	248.9	31,983	463.8
chr3a	75,567,759	3,106	3,749	1.21	18,678	4.98	1,265.9	254.1	14,929	522.0
chr3b	73,143,401	3,018	3,698	1.23	18,611	5.03	1,284.4	255.2	14,913	519.6
chr3c	73,219,076	2,984	3,610	1.21	18,275	5.06	1,272.5	251.4	14,665	552.0
chr3d	74,480,698	3,013	3,663	1.22	18,428	5.03	1,298.5	258.1	14,765	554.8
chr4a	65,602,875	3,559	4,317	1.21	21,773	5.04	1,291.0	256.0	17,456	448.7
chr4b	69,507,201	3,588	4,303	1.20	21,991	5.11	1,303.1	255.0	17,688	465.9
chr4c	64,055,324	3,395	4,102	1.21	20,785	5.07	1,297.2	256.0	16,683	473.0
chr4d	70,385,351	3,714	4,501	1.21	22,647	5.03	1,281.9	254.8	18,146	438.0
chr5a	62,029,662	4,021	4,926	1.23	26,912	5.46	1,324.3	242.4	21,986	434.7
chr5b	72,117,567	4,203	5,103	1.21	27,485	5.39	1,295.6	240.6	22,382	425.7
chr5c	61,986,087	3,716	4,507	1.21	23,647	5.25	1,272.9	242.6	19,140	445.7
chr5d	58,548,944	3,915	4,828	1.23	26,576	5.50	1,314.6	238.8	21,748	433.5
chr6a	77,839,763	3,173	3,897	1.23	20,977	5.38	1,279.0	237.6	17,080	482.8
chr6b	77,415,927	3,256	4,025	1.24	22,469	5.58	1,321.6	236.8	18,444	481.0
chr6c	78,148,598	3,313	4,089	1.23	22,093	5.40	1,287.4	238.3	18,004	458.2
chr6d	75,003,899	3,173	3,903	1.23	21,529	5.52	1,298.7	235.4	17,626	458.7
chr7a	81,487,022	4,532	5,563	1.23	30,022	5.40	1,329.8	246.4	24,459	498.7
chr7b	81,864,498	4,317	5,301	1.23	28,113	5.30	1,349.5	254.5	22,812	509.5
chr7c	81,614,575	4,553	5,573	1.22	30,886	5.54	1,339.0	241.6	25,313	497.1
chr7d	84,193,017	4,242	5,187	1.22	27,727	5.35	1,324.1	247.7	22,540	480.7

Table S9 Functional annotation summary across NCBI NR, Swiss-Prot, InterPro, eggNOG, and Pfam

Annotation Source	Annotated Genes (%)
NCBI NR	97.4
TrEMBL	97.3
TAIR10	83.3
Swiss-Prot	72.1
Pfam	81.3
eggNOG/COG	90.0
Any annotation source	>97

Table S10 Chromosomal (A) and family-level distributions (B) of miRNA loci

A	Chrom	miRNA_count
	chr1a	39
	chr1b	42
	chr1c	45
	chr1d	38
	chr2a	58
	chr2b	58
	chr2c	58
	chr2d	61
	chr3a	31
	chr3b	26
	chr3c	36
	chr3d	32
	chr4a	24
	chr4b	20
	chr4c	24
	chr4d	26
	chr4un	2
	chr5a	37
	chr5b	36
	chr5c	27
	chr5d	35
	chr5un	2
	chr6a	30
	chr6b	21
	chr6c	32
	chr6d	23
	chr6un	17
	chr7a	33
	chr7b	33
	chr7c	41
	chr7d	43
	chr7un	1

B	miRNA_family	count
	miR2593	282
	miR1520	184
	miR171_1	82
	miR1122	52
	miR159	51
	miR172	42
	miR169_2	38
	miR166	35
	miR5291	32
	miR395	23
	miR156	20
	miR393	20
	miR399	20
	miR1509	19
	miR169_6	17
	miR160	16
	miR398	13
	miR394	11
	miR396	8
	miR319	8
	miR390	7
	miR397	7
	miR2119	7
	miR162_1	4
	miR2617	4
	miR168	4
	miR171_2	4
	miR5225	4
	miR828	4
	miR408	4
	miR164	4
	miR16	2
	miR2655	2
	miR154	1

Table S11 Unique chloroplast genes

Gene	Biotype	Category	Copies	Loci
accD	protein_coding	Other protein-coding	1	80,991-82,807(-)
atpA	protein_coding	ATP synthase	1	86,373-87,900(-)
atpB	protein_coding	ATP synthase	1	5,413-6,882(-)
atpE	protein_coding	ATP synthase	1	4,974-5,364(-)
atpF	protein_coding	ATP synthase	1	87,984-88,531(-)
atpH	protein_coding	ATP synthase	1	88,887-89,132(-)
atpI	protein_coding	ATP synthase	1	89,941-90,684(-)
ccsA	protein_coding	Other protein-coding	1	17,732-18,709(+)
cemA	protein_coding	Other protein-coding	1	78,519-79,208(-)
clpP1	protein_coding	Other protein-coding	1	69,717-70,244(+)
infA	protein_coding	Other protein-coding	1	61,435-61,541(+)
matK	protein_coding	Other protein-coding	1	10,374-11,885(+)
ndhA	protein_coding	NDH (NADH dehydrogenase)	1	23,252-25,551(-)
ndhB	protein_coding	NDH (NADH dehydrogenase)	1	44,974-47,191(+)
ndhC	protein_coding	NDH (NADH dehydrogenase)	1	3,187-3,549(-)
ndhD	protein_coding	NDH (NADH dehydrogenase)	1	18,974-20,467(-)
ndhE	protein_coding	NDH (NADH dehydrogenase)	1	21,140-21,445(-)
ndhF	protein_coding	NDH (NADH dehydrogenase)	1	13,926-16,160(-)
ndhG	protein_coding	NDH (NADH dehydrogenase)	1	21,665-22,195(-)
ndhH	protein_coding	NDH (NADH dehydrogenase)	1	25,553-26,734(-)
ndhI	protein_coding	NDH (NADH dehydrogenase)	1	22,686-23,167(-)
ndhJ	protein_coding	NDH (NADH dehydrogenase)	1	1,879-2,355(-)
ndhK	protein_coding	NDH (NADH dehydrogenase)	1	2,510-3,144(-)
pafI	protein_coding	Other protein-coding	1	118,095-120,090(-)
pafII	protein_coding	Other protein-coding	1	79,596-80,039(-)
pbf1	protein_coding	Other protein-coding	1	67,249-67,380(+)
petA	protein_coding	Cytochrome b6/f	1	77,353-78,315(-)
petB	protein_coding	Cytochrome b6/f	1	65,336-65,981(-)
petD	protein_coding	Cytochrome b6/f	1	63,918-64,396(-)
petG	protein_coding	Cytochrome b6/f	1	74,481-74,594(-)
petL	protein_coding	Cytochrome b6/f	1	74,746-74,841(-)
petN	protein_coding	Cytochrome b6/f	1	104,036-104,125(+)
psaA	protein_coding	Photosystem I	2	112,858-114,141(-);114,929-117,179(-)
psaB	protein_coding	Photosystem I	2	112,699-114,903(-);115,073-116,962(-)
psaC	protein_coding	Photosystem I	1	20,598-20,843(-)
psaI	protein_coding	Photosystem I	1	80,402-80,506(-)
psaJ	protein_coding	Photosystem I	1	73,442-73,568(-)
psbA	protein_coding	Photosystem II	2	12,421-13,482(+);13,796-108,554(+)

psbB	protein_coding	Photosystem II	1	67,747-69,273(-)
psbC	protein_coding	Photosystem II	1	108,952-110,337(+)
psbD	protein_coding	Photosystem II	1	107,907-108,968(+)
psbE	protein_coding	Photosystem II	1	75,783-76,034(+)
psbF	protein_coding	Photosystem II	1	76,044-76,163(+)
psbH	protein_coding	Photosystem II	1	66,898-67,130(-)
psbI	protein_coding	Photosystem II	1	84,229-84,339(+)
psbJ	protein_coding	Photosystem II	1	76,442-76,564(+)
psbK	protein_coding	Photosystem II	1	83,715-83,900(+)
psbL	protein_coding	Photosystem II	1	76,186-76,302(+)
psbM	protein_coding	Photosystem II	1	104,982-105,086(-)
psbT	protein_coding	Photosystem II	1	67,446-67,553(-)
psbZ	protein_coding	Photosystem II	1	110,962-111,150(+)
rbcL	protein_coding	RuBisCO large subunit	1	7,625-9,052(+)
rpl14	protein_coding	Ribosomal protein, large subunit	1	59,989-60,357(+)
rpl16	protein_coding	Ribosomal protein, large subunit	1	59,464-59,866(+)
rpl2	protein_coding	Ribosomal protein, large subunit	1	55,366-56,891(+)
rpl20	protein_coding	Ribosomal protein, large subunit	1	71,514-71,863(+)
rpl22	protein_coding	Ribosomal protein, large subunit	1	57,363-57,539(+)
rpl23	protein_coding	Ribosomal protein, large subunit	1	55,064-55,345(+)
rpl32	protein_coding	Ribosomal protein, large subunit	1	16,779-16,931(+)
rpl33	protein_coding	Ribosomal protein, large subunit	1	72,733-72,933(-)
rpl36	protein_coding	Ribosomal protein, large subunit	1	61,725-61,838(+)
rpoA	protein_coding	RNA polymerase	1	62,724-63,725(+)
rpoB	protein_coding	RNA polymerase	1	99,069-102,281(-)
rpoC1	protein_coding	RNA polymerase	1	96,233-99,042(-)
rpoC2	protein_coding	RNA polymerase	1	91,898-96,040(-)
rps11	protein_coding	Ribosomal protein, small subunit	1	62,226-62,642(+)
rps12	protein_coding	Ribosomal protein, small subunit	2	43,996-44,253(+);70,644-70,757(+)
rps14	protein_coding	Ribosomal protein, small subunit	1	112,272-112,574(-)
rps15	protein_coding	Ribosomal protein, small subunit	1	26,832-27,104(-)
rps18	protein_coding	Ribosomal protein, small subunit	1	72,127-72,456(-)
rps19	protein_coding	Ribosomal protein, small subunit	1	57,073-57,346(+)
rps2	protein_coding	Ribosomal protein, small subunit	1	90,901-91,611(-)
rps3	protein_coding	Ribosomal protein, small subunit	1	57,524-58,189(+)
rps4	protein_coding	Ribosomal protein, small subunit	1	120,636-121,244(-)
rps7	protein_coding	Ribosomal protein, small subunit	1	44,309-44,776(+)
rps8	protein_coding	Ribosomal protein, small subunit	1	60,910-61,314(+)
ycf1	protein_coding	YCF/other	1	27,544-33,071(-)
ycf2	protein_coding	YCF/other	1	48,343-54,127(+)
rrn16	rRNA	rRNA	1	40,332-41,823(-)

rrn23	rRNA	rRNA	2	35,150-35,721(-);35,735-37,958(-)
rrn4.5	rRNA	rRNA	1	34,948-35,051(-)
rrn5	rRNA	rRNA	1	34,639-34,759(-)
trnA-UGC	tRNA	tRNA	1	38,908-39,012(-)
trnC-GCA	tRNA	tRNA	1	103,516-103,586(+)
trnD-GUC	tRNA	tRNA	1	105,499-105,572(-)
trnE-UUC	tRNA	tRNA	1	106,160-106,232(-)
trnF-GAA	tRNA	tRNA	1	1,115-1,187(+)
trnG-GCC	tRNA	tRNA	1	111,822-111,892(+)
trnH-GUG	tRNA	tRNA	1	13,642-13,715(+)
trnL-CAA	tRNA	tRNA	1	54,691-54,771(-)
trnL-UAA	tRNA	tRNA	1	403-890(+)
trnL-UAG	tRNA	tRNA	1	17,534-17,613(+)
trnM-CAU	tRNA	tRNA	3	4,795-4,867(+);48,086-48,159(-);112,037-112,110(-)
trnN-GUU	tRNA	tRNA	1	33,688-33,759(+)
trnP-UGG	tRNA	tRNA	1	74,016-74,089(+)
trnQ-UUG	tRNA	tRNA	1	83,102-83,173(-)
trnR-ACG	tRNA	tRNA	1	34,331-34,404(-)
trnR-UCU	tRNA	tRNA	1	86,165-86,236(+)
trnS-GCU	tRNA	tRNA	1	84,477-84,563(-)
trnS-GGA	tRNA	tRNA	1	120,348-120,435(+)
trnS-UGA	tRNA	tRNA	1	110,513-110,601(-)
trnT-GGU	tRNA	tRNA	1	106,699-106,772(+)
trnT-UGU	tRNA	tRNA	1	121,540-121,612(-)
trnV-GAC	tRNA	tRNA	1	42,171-42,242(-)
trnW-CCA	tRNA	tRNA	1	74,270-74,343(+)
trnY-GUA	tRNA	tRNA	1	106,002-106,085(-)

Table S12 Unique mitochondrial genes

Gene	Class	Category	Loci	Evidence
orf100a	hypothetical_ORF	Hypothetical ORF	109,665-109,953(-)	blatX
orf100c	hypothetical_ORF	Hypothetical ORF	150,405-150,515(+)	blatX
orf101a	hypothetical_ORF	Hypothetical ORF	223,457-223,762(+)	blatX
orf101b	hypothetical_ORF	Hypothetical ORF	241,359-241,688(-)	blatX
orf103a	hypothetical_ORF	Hypothetical ORF	127,384-127,690(+)	blatX
orf103d	hypothetical_ORF	Hypothetical ORF	298,991-299,139(-)	blatX
orf104a	hypothetical_ORF	Hypothetical ORF	99,173-99,224(-);300,840-300,915(+)	blatX
orf104b	hypothetical_ORF	Hypothetical ORF	275,228-275,539(-)	blatX
orf106a	hypothetical_ORF	Hypothetical ORF	20,746-21,071(-)	blatX
orf109	hypothetical_ORF	Hypothetical ORF	278,474-278,785(+)	blatX
orf110b	hypothetical_ORF	Hypothetical ORF	70,690-71,025(-)	blatX
orf110c	hypothetical_ORF	Hypothetical ORF	130,877-172,049(-)	blatX
orf114a	hypothetical_ORF	Hypothetical ORF	202,531-202,674(-)	blatX
orf129	hypothetical_ORF	Hypothetical ORF	63,006-63,386(-);148,248-148,325(+)	blatX
orf139	hypothetical_ORF	Hypothetical ORF	298,770-299,139(-)	blatX
orf150	hypothetical_ORF	Hypothetical ORF	285,477-300,230(+)	blatX
orf151	hypothetical_ORF	Hypothetical ORF	249,178-249,209(+)	blatX
orf160a	hypothetical_ORF	Hypothetical ORF	190,328-190,425(-)	blatX
orf160b	hypothetical_ORF	Hypothetical ORF	171,086-171,139(-)	blatX
orf172	hypothetical_ORF	Hypothetical ORF	171,881-172,189(-)	blatX
orf178	hypothetical_ORF	Hypothetical ORF	297,872-298,372(+)	blatX
orf202	hypothetical_ORF	Hypothetical ORF	111,082-111,697(-)	blatX
orf241	hypothetical_ORF	Hypothetical ORF	181,100-181,382(+)	blatX
orf261	hypothetical_ORF	Hypothetical ORF	54,008-54,040(-)	blatX
orf271	hypothetical_ORF	Hypothetical ORF	314,598-314,743(+)	blatX
orf287	hypothetical_ORF	Hypothetical ORF	110,137-154,287(-)	blatX
atp1	protein_coding	Complex V (ATP synthase)	3,280-4,786(+);3,280-4,795(+);233,744-234,829(-)	blatX
atp4	protein_coding	Complex V (ATP synthase)	170,561-171,139(-)	blatX
atp6	protein_coding	Complex V (ATP synthase)	289,292-289,998(+);289,292-290,002(+)	blatX
atp8	protein_coding	Complex V (ATP synthase)	143,780-144,262(-);148,318-162,978(-)	blatX
atp9	protein_coding	Complex V (ATP synthase)	132,675-132,899(+)	blatX
ccmB	protein_coding	Cytochrome c maturation	172,808-173,428(-)	blatX
ccmC	protein_coding	Cytochrome c maturation	60,649-61,389(-)	blatX
ccmFc	protein_coding	Cytochrome c maturation	23,498-25,709(-)	blatX
ccmFn	protein_coding	Cytochrome c maturation	296,651-298,372(+)	blatX
cob	protein_coding	Complex III (cyt b)	47,240-48,409(+)	blatX
cox1	protein_coding	Complex IV (cytochrome c oxidase)	307,445-309,028(+)	blatX

cox2	protein_coding	Complex IV (cytochrome c oxidase)	161,986-162,730(-);195,466-195,681(+)	blatX
cox3	protein_coding	Complex IV (cytochrome c oxidase)	148,891-149,688(+)	blatX
matR	protein_coding	Maturase	93,024-94,984(+)	blatX
mttB	protein_coding	Tat/Mtt protein export	256,201-256,927(+)	blatX
nad1	protein_coding	Complex I (NADH dehydrogenase)	85,875-95,761(+);85,875-311,866(+);310,336-311,866(+)	blatX
nad2	protein_coding	Complex I (NADH dehydrogenase)	113,105-117,294(+);113,105-313,669(+);196,381-313,669(+)	blatX
nad3	protein_coding	Complex I (NADH dehydrogenase)	314,666-315,022(+)	blatX
nad4	protein_coding	Complex I (NADH dehydrogenase)	11,378-19,931(-)	blatX
nad4L	protein_coding	Complex I (NADH dehydrogenase)	171,324-171,626(-)	blatX
nad5	protein_coding	Complex I (NADH dehydrogenase)	220,824-269,719(+)	blatX
nad6	protein_coding	Complex I (NADH dehydrogenase)	232,412-233,029(-)	blatX
nad7	protein_coding	Complex I (NADH dehydrogenase)	72,325-78,778(+)	blatX
nad9	protein_coding	Complex I (NADH dehydrogenase)	120,369-120,877(+)	blatX
rpl16	protein_coding	Ribosomal protein, large	50,154-50,678(-)	blatX
rpl5	protein_coding	Ribosomal protein, large	45,062-45,613(+)	blatX
rps1	protein_coding	Ribosomal protein, small	218,744-219,256(+)	blatX
rps12	protein_coding	Ribosomal protein, small	315,067-315,444(+)	blatX
rps14	protein_coding	Ribosomal protein, small	45,617-45,919(+)	blatX
rps3	protein_coding	Ribosomal protein, small	50,560-54,040(-)	blatX
rps4	protein_coding	Ribosomal protein, small	78,952-79,992(+)	blatX
rrn18	rRNA	rRNA	1-396(+);237,713-238,188(-);237,713-238,254(-);238,255-239,377(-);238,255-239,508(-);319,761-321,014(+);319,892-321,014(+)	blatN
rrn26	rRNA	rRNA	186,785-187,174(+);186,989-189,923(+);187,180-189,924(+)	blatN
rrn5	rRNA	rRNA	577-692(+);237,417-237,532(-)	blatN
trnC-GCA	tRNA	tRNA	110,422-110,492(+)	tRNAscan-SE_v2
trnD-GUC	tRNA	tRNA	21,559-21,632(+)	tRNAscan-SE_v2
trnE-UUC	tRNA	tRNA	39,836-39,907(-)	tRNAscan-SE_v2
trnF-GAA	tRNA	tRNA	105,379-105,442(-);300,305-300,378(-)	tRNAscan-SE_v2
trnG-GCC	tRNA	tRNA	275,240-275,311(+)	tRNAscan-SE_v2
trnH-GUG	tRNA	tRNA	34,719-34,792(-)	tRNAscan-SE_v2
trnK-UUU	tRNA	tRNA	127,338-127,410(-)	tRNAscan-SE_v2
trnM-CAU	tRNA	tRNA	38,894-38,966(-);190,295-190,368(+);317,247-317,320(-)	tRNAscan-SE_v2
trnN-GUU	tRNA	tRNA	112,335-112,406(+)	tRNAscan-SE_v2
trnP-UGG	tRNA	tRNA	299,976-300,050(-)	tRNAscan-SE_v2
trnQ-UUG	tRNA	tRNA	277,690-277,761(+)	tRNAscan-SE_v2
trnS-GCU	tRNA	tRNA	300,732-300,819(-)	tRNAscan-SE_v2
trnT-UGU	tRNA	tRNA	269,833-269,911(+)	tRNAscan-SE_v2

trnW-CCA	tRNA	tRNA	241,295-241,368(-)	tRNAscan-SE_v2
trnY-GUA	tRNA	tRNA	112,546-112,628(+)	tRNAscan-SE_v2
trnnull-NNN	tRNA	tRNA	232,367-232,426(-)	tRNAscan-SE_v2

Table S13 Significantly enriched GO terms among strongly biased allele-specific expression groups under water stress

Condition	GO ID	Term name	Namespace	Study genes	Background genes	adjusted p-value
Waterlogging	GO:0042545	Cell wall modification	BP	9	146	0.0167
Drought	GO:0004497	Monooxygenase activity	MF	10	232	0.0259
Drought	GO:0044550	Secondary metabolite biosynthetic process	BP	11	304	0.0361
Drought	GO:0016709	Oxidoreductase activity, acting on paired donors with NAD(P)H as donor and incorporation of oxygen	MF	8	156	0.0361

BP, biological process; MF, molecular function
